# Drug-Nutraceutical Co-Crystal and Salts for Making New and Improved Bi-Functional Analgesics

**DOI:** 10.3390/pharmaceutics12121144

**Published:** 2020-11-26

**Authors:** Oli Abate Fulas, André Laferrière, Ghada Ayoub, Dayaker Gandrath, Cristina Mottillo, Hatem M. Titi, Robin S. Stein, Tomislav Friščić, Terence J. Coderre

**Affiliations:** 1 Department of Anesthesia, McGill University, 3655 Promenade Sir William Osler, Montreal, QC H3G 1Y6, Canada; oli.fulas@mail.mcgill.ca (O.A.F.); andre.laferriere@mcgill.ca (A.L.); 2 Department of Chemistry, McGill University, 801 Sherbrooke Street W, Montreal, QC H3A 0B8, Canada; ghada.ayoub@mail.mcgill.ca (G.A.); dayaker.gandrath@intellisynrd.com (D.G.); cristina.mottillo@mail.mcgill.ca (C.M.); hatem.titi@mcgill.ca (H.M.T.); robin.stein@mcgill.ca (R.S.S.)

**Keywords:** neuropathic pain, CRPS, mechanochemistry, co-crystal, topical analgesics, vasodilators, antioxidants

## Abstract

The discovery and development of effective analgesics is greatly lagging behind the steadily rising prevalence of chronic pain. Currently prescribed analgesics for chronic pain are lacking in efficacy mainly due to their narrowly-targeted mechanism of action. Driving neuronal hyperexcitability that underlies symptoms of chronic pain are multiple non-neuronal processes, among which are tissue hypoxia and oxidative stress. Here we demonstrate the design, synthesis, and activity of new multi-component bi-functional analgesic crystalline solids, co-crystals, and salts, based on pairing of vasodilatory anti-hypoxic drugs pentoxifylline, clonidine and linsidomine with antioxidant nutraceuticals protocatechuic acid, α-lipoic acid, and caffeic acid. After validation, chemical and structural characterization of these novel salts and co-crystals, topical formulations of the products were tested in a rat model of complex regional pain syndrome. Analgesic effects achieved with the salts and co-crystal exceeded the efficacy and/or potency of constituent compounds indicating that more effective, advanced analgesics can readily be developed by careful pairing of compounds that simultaneously target multiple neural and non-neural processes driving chronic pain.

## 1. Introduction

Chronic pain is a highly prevalent, expensive to treat condition that causes physical disability and psychological debilitation to nearly 22% of the general population [1]. Its complex pathophysiology that involves interdependent neuronal, immune and vascular maladaptive processes has made it difficult to manage despite extensive research [2,3]. Currently available drugs fail to provide optimal analgesia due to limited efficacy and dose limiting side-effects [4]. One reason for the limited efficacy exhibited by most analgesics in clinical use is their narrowly directed mechanism of action that impacts a limited aspect of the disease process [5]. This can be circumvented by the introduction of broader-purposed multi-component analgesics.

The past decade has witnessed the rapid development of designs and uses of advanced multi-component pharmaceutical solids. These are often based on association of an active pharmaceutical ingredient (API) with additional counter molecules in order to provide a novel and unique crystalline material whose structure and physicochemical properties are distinct from those of pure component solid forms [6]. Two most significant types of such advanced pharmaceutical materials are salts, typically resulting from a proton transfer process between different components of the material [7], and cocrystals, in which the components of the material are assembled in a predictable fashion through principles of supramolecular chemistry and molecular recognition [8,9]. Whereas salt formation (salification) has been extensively used in the development of API solid forms and formulations [7,10], pharmaceutical co-crystals have only relatively recently emerged as a means to modify solid-state properties and bioavailability of drug molecules with no ionizable sites [8,9,11]. The design of pharmaceutical co-crystals is typically based on an API component, with one or more co-crystal formers (co-formers) that belong to the “Generally Regarded As Safe” (GRAS) list of compounds [11,12,13]. This modular design has enabled the design and synthesis of API forms with improved or modified physicochemical properties, such as melting point, tabletability, solubility, dissolution rate, bioavailability and stability [14,15,16,17,18,19,20]. A considerably less explored design of pharmaceutical co-crystals involves the use of one or more API molecules as components [21,22], designed for the primary purpose of targeting multiple disease processes of a given pathology [23,24]. This application is even less explored in the field of analgesic development for chronic pain [25].

**Figure 1:**
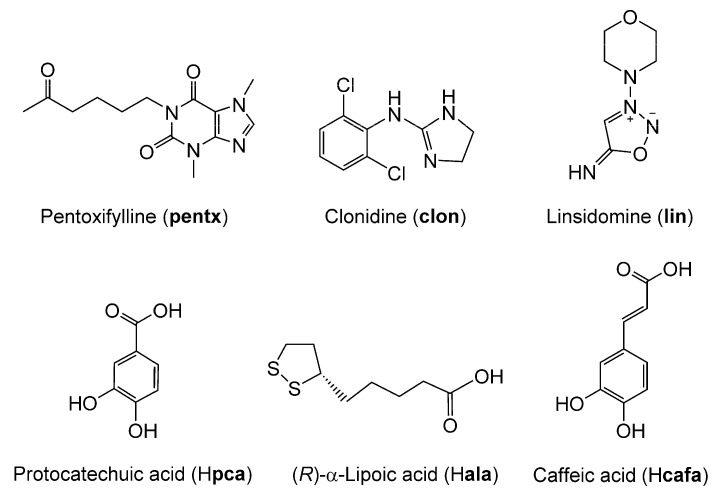
Molecular structures of pentoxifylline (**pentx**), clonidine (**clon**), linsidomine (**lin**), protocatechuic acid (H**pca**), (*R*)-α-lipoic acid (H**ala**) and caffeic acid (H**cafa**).

Here, we report the design and synthesis of advanced, bi-functional therapeutic co-crystal and salts made by combining vasodilatory anti-hypoxic drugs pentoxifylline (**pentx**), clonidine (**clon**), and linsidomine (**lin**), with anti-oxidant nutraceuticals protocatechuic acid (H**pca**), α-lipoic acid (H**ala**) and caffeic acid (H**cafa**), respectively. Our design is based on the observation that, in addition to their complementary mechanism of action that alleviates chronic pain symptoms, these two groups of compounds exhibit chemical structures that would be very suited for mutual co-crystallization and/or salt formation. Pentoxifylline is a methyl xanthine with an aromatic nitrogen in the imidazole ring and two carbonyl groups that should promote co-crystal formation with carboxylic acids through hydrogen bonding (Figure 1) [26]. In contrast, clonidine is an imidazoline derivative that contains a strongly basic guanidine group suitable for salt formation with carboxylic acids. Similarly, linsidomine is a morpholine derivative exhibiting a highly basic imine group that should allow for salt formation in presence of a carboxylic acid (Figure 1). All the nutraceutical antioxidants protocatechuic acid, α-lipoic acid and caffeic acid (3-(3,4-Dihydroxyphenyl)-2-propenoic acid) possess a carboxylic acid group capable of acting as a hydrogen bond donor for co-crystallization, or proton donors for salt formation (Figure 1).

The syntheses of these salt and co-crystal materials were aimed at simultaneously relieving the pain inducing hypoxia and oxidative stress seen in peripheral tissue affected by chronic pain conditions including complex regional pain syndrome (CRPS) and peripheral neuropathic pain [27,28]. In a rat model of CRPS, we demonstrate the augmentation of analgesic efficacy and/or potency achieved by co-crystallization and salt formation between **pentx**, **clon**, and **lin** and the nutraceutical antioxidants H**pca**, H**ala**, and H**cafa**.

## 2. Materials and Methods

### 2.1. Materials

Pentoxifylline (>97%) and caffeic acid (>98%) were obtained from Sigma-Aldrich (St. Louis, MO, USA). Linsidomine chloride (>99%), clonidine hydrochloride (>98%), and protocatechuic acid (>98%) were purchased from Cayman Chemicals (Ann Arbor, MI, USA) and (*R*)-α-lipoic acid (>98%) from TCI chemicals (Portland, OR, USA). Acetonitrile (>99.5%) and diethyl ether (>99%) were obtained from Fisher Chemical (Saint-Laurent, QC, Canada) while ethanol (95%) was purchase from Commercial Alcohols (Toronto, ON, Canada).

### 2.2. Synthesis of Salts/Co-Crystals of Drugs

Synthesis of **clon**, **lin**, and **pentx** co-crystals/salts with the nutraceuticals H**ala**, H**cafa**, and H**pca** was performed by mechanochemical liquid-assisted grinding (LAG) [29,30]. This was achieved. by milling in the presence of a small, near-stoichiometric amount of a liquid additive (measured by *η* [30], i.e., the ratio of added liquid volume to the weight of reactant mixture, in μL/mg) in order to improve the synthetic efficiency and provide a well-defined crystalline product. In particular, prior work has demonstrated that, whereas neat (dry) milling of molecular solids often leads to amorphization and poor reactivity, the addition of a liquid in the *η*-range below ca. 2 μL/mg leads to rapid and often quantitative cocrystal formation, independent of the solubility of each component [29,30]. Samples were placed in 15 mL volume polytetrafluoroethylene (PTFE) jars, along with a small amount of ethanol (EtOH) and a single stainless-steel ball of 10 mm diameter (4 g weight). Ball milling was done for 30 min in a Retsch MM400 shaker mill (Retsch GmbH, Hann, Germany) operating at 30 Hz. The analgesic components **clon** and **pentx** were paired with equimolar quantities of H**ala** and H**pca**, respectively. For the pair **clon** and H**ala**, LAG was performed in the presence of 50 μL EtOH (*η* = 0.47 μL/mg) as the liquid additive, while 100 μL EtOH (*η* = 0.46 μL/mg) was used for the pair **pentx** and H**pca**. The solid **clon** was first obtained from the commercially available hydrochloride salt (H**clon**^+^Cl^−^) form following the mechanochemical procedure described below (vide infra).

Due to the instability of the **lin** free base, the salt analgesic (H**lin**^+^)(**cafa**^−^) was synthesized by a mechanochemical anion exchange reaction. For this purpose, solid H**cafa** was first reacted with an equimolar quantity of sodium hydroxide (NaOH) by neat grinding for 30 min in a stainless steel jar, using two stainless steel balls of 7 mm diameter (1.3 g weight each) and a Retsch MM400 shaker mill operating at 30 Hz. The product, which was confirmed to be sodium caffeate (Na^+^**cafa**^−^) using crystallographic and spectroscopic methods, was then used for mechanochemical ion exchange with (H**lin**^+^Cl^−^), conducted by LAG in the presence of 10 µL EtOH, in a PTFE-based milling assembly. Milling for 45 min using a Retsch MM400 shaker mill operating at 30 Hz produced (H**lin**^+^)(**cafa**^−^) along with NaCl.

#### Purification of Clonidine-Hydrochloride

The API **clon** is commercially available as clonidine hydrochloride (H**clon**^+^Cl^−^) from which the free base was obtained by treatment with a concentrated solution of potassium hydroxide in deionized water. The reaction yielded water-soluble KCl and the **clon** free base which readily precipitated out of solution. The resulting solid **clon** was separated and dried by vacuum filtration, and its composition confirmed via nuclear magnetic resonance (NMR) in CDCl_3_ solution and Fourier-transform infrared attenuated total reflectance (FTIR-ATR) spectroscopy.

### 2.3. Validation and Characterization Experiments on Synthesized Salts and Co-Crystals

#### 2.3.1. FTIR-ATR Spectroscopy

Measurements were performed on a Spectrum TWO FTIR with single bounce diamond ATR from Perkin Elmer (Waltham, MA, USA) in the range of 4000–400 cm^−1^ and with resolution of 1 cm^−1^.

#### 2.3.2. Powder X-ray Diffraction (PXRD)

PXRD experiments on starting materials and reaction products were conducted on a Bruker D2 phaser (Bruker-AXS, Madison, WI, USA) equipped with Cu*K*α X-ray source and a Nickel filter. Data was collected in the 2*θ*-range of 4–40° with increment of 0.05°.

#### 2.3.3. Single Crystal X-ray Diffraction (SCXRD)

Single crystals used for single crystal X-ray diffraction (SCXRD) were grown at room temperature by slow evaporation. The solvent used for the (**pentx**)(H**pca)** co-crystal was a mixture of EtOH and acetonitrile, and for the (H**clon^+^**)(**ala^−^**) salt, it was diethyl ether.

Single crystal X-ray diffraction data for all compounds were collected on a Bruker D8 Advance diffractometer (Bruker-AXS, Madison, WI, USA) with a Photon 100 CMOS area detector and an IμS microfocus X-ray source (Bruker AXS). X-ray diffraction experiments on single crystals of the (**pentx**)(H**pca)** co-crystal and the (H**clon^+^**)(**ala^−^**) salt were conducted using Cu*K*α radiation. Single crystals were often found to readily lose solvent upon exposure to air, and were coated with Paratone oil (Hampton Research, Aliso Viejo, CA, USA) during X-ray single crystal data collection.

Unit cell determination, data collection, data reduction, and correction for absorption were all conducted using the Apex3 software suite (Bruker AXS, Madison, WI, USA). The crystal structures were solved by an iterative dual space approach as implemented in SHELXT [31]. Non-hydrogen atoms were located from the difference map and refined anisotropically. Hydrogen atoms bonded to carbon atoms were placed in calculated positions. All hydrogen atoms coordination and thermal parameters were constrained to ride on the carrier atoms. Both the co-crystal and the salt crystals were found to be twinned and the structure of (**pentx**)(H**pca)** was treated by CELL NOW (Version 2008-2, Bruker AXIS Inc., WI, USA), which enabled us to identify three domains of non-merohedral twinning. The domains are rotated by 180° and by 3.4°, with the main domain adopting the orientation matrix of (1 0 0, 0 −1 0, −0.64 0 −1). On the other hand, the (H**clon^+^**)(**ala^−^**) was resolved as racemic twin using the inversion orientation matrix of (−1 0 0, 0 −1 0, 0 0 −1).

#### 2.3.4. Thermogravimetric Analysis (TGA) and Differential Scanning Calorimetry (DSC)

TGA was performed using TGA Q500 (TA Instruments, New Castle, DE, USA). Approximately 5 mg of the samples were heated over a temperature range of 25 °C to 600 °C while being purged with a flow of air and nitrogen gas throughout the experiment. The resulting thermograms were analyzed with TA Universal Analysis software (version 6.7, TA Instruments, New Castle, DE, USA). The DSC experiments were performed on a DSC2500 (TA instruments Ltd., New Castle, DE, USA), under a stream (50 mL min^−1^) of nitrogen gas. Samples (2 mg) were placed into hermetically-sealed aluminum pans, which were first cooled to −20 °C and left in isotherm for 1 min before heating up the samples to 200 °C, in a heating rate of 5 °C s^−1^.

#### 2.3.5. Nuclear Magnetic Resonance Spectroscopy (NMR)

Solution ^1^H-NMR spectra (300 MHz) were recorded on Varian Mercury 300 MHz NMR spectrometer (Varian Inc., Palo Alto, CA, USA) for clonidine. Chemical shifts are reported relative to CDCl_3_ (δ 7.26 ppm) for ^1^H-NMR spectra. The ^1^H NMR data are presented as follows: Chemical shift, multiplicity and integration.

Solid state NMR (ssNMR) was performed using a Varian 400 MHz VNMRS wide bore spectrometer (Varian Inc., Palo Alto, CA, USA) operating at a ^1^H frequency of 399.76 MHz, a ^13^C frequency of 100.53 MHz. CPMAS ^13^C spectra were acquired using a 4 mm double-resonance probe (Varian Inc., Palo Alto, CA, USA).

Spectra of (Na^+^**cafa**^−^) and (H**lin**^+^Cl^−^) were acquired under spinning at 5 kHz. That of (H**lin**^+^)(**cafa**^−^) was acquired at 8 kHz, and that of H**cafa** was acquired at 13 kHz spinning. The spectrum of (H**lin**^+^Cl^−^) was compared with a CPTOSS spectrum (not shown) to identify spinning sidebands. A recycle delay of 4 s was used for H**cafa**, while a 5 s delay was used for (Na^+^**cafa**^−^) and (H**lin**^+^Cl^−^), and 6 s for (H**lin**^+^)(**cafa**^−^). A contact time of 3.5 ms was used for H**cafa** and (Na^+^**cafa**^−^), 1.5 ms was used for (H**lin**^+^Cl^−^), and 3.0 ms was used for (H**lin**^+^)(**cafa**^−^). A total of 128 transients were acquired of (Na^+^**cafa**^−^), 1280 of (H**lin**^+^Cl^−^), 172 of (H**lin**^+^)(**cafa**^−^), and 8192 of H**cafa**. The ^13^C spectra were referenced to TMS using the carbonyl signal of glycine at 176.04 ppm.

### 2.4. Formulation of Drugs into Topical Ointments

Topical drugs were formulated into ointment-type preparations using a composite, water-soluble polyethylene glycol (PEG) base system consisting of 40% carbowax (PEG 3350) and 60% PEG 400 (both from Sigma Aldrich, St. Louis, MO, USA). The required amounts of the active ingredients were first weighted and then added to the molten base and mixed. The vehicle treatment consisted of the same water-miscible base ointment without the addition of the active drugs.

### 2.5. Generation of the Rat Model of CRPS

All experimental manipulations in experimental animals were approved by the McGill University Animal Ethics committee in conformity to CCAC regulations (protocol application # 20177877, approved on 7 March 2017). A total of 35 rats were used in these experiments and all were housed two per cage in a temperature (21–25 °C) and humidity-controlled room on a 12 h light/dark cycle beginning at 7:00 AM with ad libitum access to rodent chow and water.

A rat model of CRPS was prepared by inducing prolonged hind paw ischemia and subsequent reperfusion, previously described by Coderre et al. as chronic post-ischemia pain (CPIP) [32]. In short, male Long Evans rats (300–400 g; Charles River, QC, Canada) were anesthetized over a 3-h period with a bolus (55 mg/kg, intraperitoneally [i.p.]) followed by chronic i.p. infusion (0.15 mL/h) of sodium pentobarbital (Ceva Sante Animale, Libourne, France) for 2 h. Following induction of anesthesia, a Nitrile 70 Durometer O-ring (O-rings West, Seattle, WA, USA) with an internal diameter of 5.5-mm was slipped around the rat’s left hind limb proximal to the ankle joint to effect a complete blockade of arterial blood flow [33]. The ring was left in place for three hours, and the rats recovered from anesthesia 30 to 60 min following reperfusion.

### 2.6. Mechanical Sensitivity Testing

As a measure of mechanical sensitivity, paw withdrawal threshold (PWT), was tested on plantar surface of the ipsilateral hind paw of the CPIP rats using von Frey filaments (Semmes-Weinstein von Frey hairs, Stoelting, Wood Dale, IL, USA). The rats were first habituated for 30 min in the test chamber. Nylon monofilaments were applied in either ascending (after negative response) or descending (after positive response) force as necessary to determine the filament closest to the threshold of response. Each filament was applied for 10 s or until a flexion reflex occurred. The minimum stimulus intensity was 0.25 g and the maximum was 15 g. Based on the response pattern, and the force of the final filament (5th stimulus after first direction change), the 50% threshold (grams) was calculated as (10 ^[Xf + kδ]^)/10,000, where X_f_ = value (in log units) of the final von Frey hair used, k = value for the pattern of positive/negative responses and δ = mean difference in log unit between stimuli (here, δ = 0.224, for more detail see Chaplan et al., 1994) [34]. PWT was assessed before ischemia/reperfusion injury, and prior to and following topical treatments.

### 2.7. Statistics on Animal Behavioral Data

Analysis of animal behavioral data was performed using the statistical software Statistica (version 6, StatSoft Inc, Tulsa, OK, USA). Time course measurements of PWTs after vehicle and drug administration were subjected to a repeated measure analysis of variance (ANOVA). Pairwise post-hoc comparisons were performed between PWTs obtained from drug and vehicle treatment groups measured at matching post-treatment times using Tukey’s HSD test.

The cumulative anti-allodynic effect measured over 180-min time course experiments was assessed by calculating the area under the curve (AUC) of PWT elevations plotted post-topical application. Comparisons of different drug doses versus vehicle were performed using repeated measures ANOVA followed by Tukey’s HSD test.

Dose response curves for comparison of anti-allodynic potency were plotted on a semi-log scale with the amount of drug used per application on the *X*-axis and the AUC of the 180-min PWTs measured or change in PWTs obtained post-drug application on the *Y*-axis. The linear regressions of the dose-response curves were then calculated, and their difference analyzed using 1-way ANOVA. Differences in anti-allodynic potency are stated in terms of the shift in the x-intercept of the regression line of each drug’s dose-response curve.

## 3. Results

### 3.1. Validation and Characterization of Synthesized Co-Crystal and Salts

#### 3.1.1. Co-Crystal of Pentoxifylline and Protocatechuic Acid

**Figure 2:**
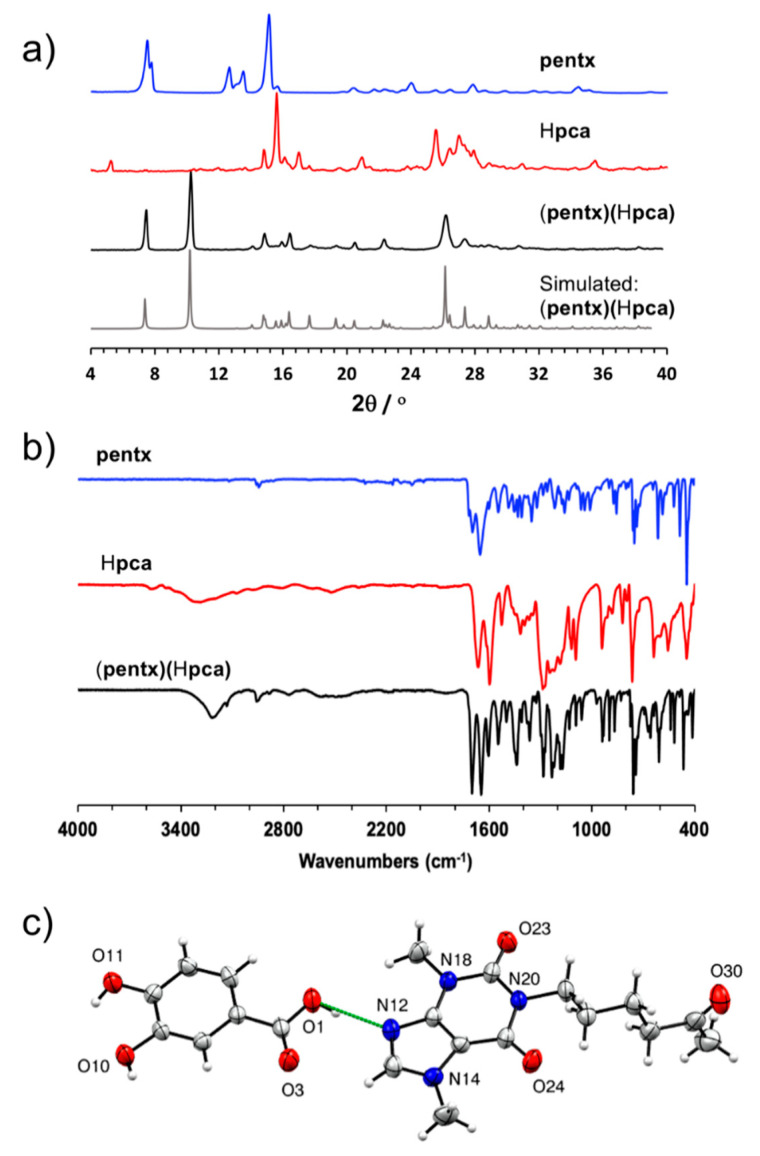
(**a**) Comparison of powder X-ray diffraction (PXRD) patterns, as well as (**b**) FTIR spectra for **pentx**, H**pca**, and the (**pentx**)(H**pca)** co-crystal (**c**) The asymmetric unit in (**pentx**)(H**pca)**, in which the (**pentx**)(H**pca**) entities are held together through hydrogen bond depicted in dashed green line.

The co-crystal of pentoxifylline and protocatechuic acid (**pentx**)(H**pca**) was first obtained mechanochemically by LAG of the two solid components in the presence of EtOH. Co-crystal formation was evidenced by PXRD analysis of the solid mixture after milling, which revealed the complete disappearance of signals belonging to the starting materials and the appearance of new Bragg reflections at 2*θ*-values of 10.3°, 16.5°, and 22.3° that are not present in the PXRD patterns of starting materials (Figure 2a). Co-crystal formation also resulted in changes to the FTIR-ATR spectrum compared to the individual solid components and, notably, the C=O stretching band was found to shift slightly from 1274 cm^−1^ in the protocatechuic acid spectrum to 1283 cm^−1^ in (**pentx**)(H**pca**), implying a change in the hydrogen bonding pattern that would be consistent with co-crystallization (Figure 2b and Figure S12 in Supplementary Materials). Thermal analysis of (**pentx**)(H**pca**) was performed in the range from −20 °C to 200 °C, which revealed a sharp endothermic event at 148 °C, which is consistent with melting (see Figure S4 in Supplementary Materials). Importantly, the DSC thermograms of pure solids **pentx** and H**pca** exhibit melting point signals at 107 °C and 132 °C, which confirms that (**pentx**)(H**pca**) is not a physical mixture of the starting materials, as well as that it exhibits a higher thermal stability in the solid state (see Figure S4 in Supplementary Materials).

Single crystals of the (**pentx**)(H**pca)** co-crystal were obtained by slow evaporation from a solution of 1 mL in a mixture of EtOH and acetonitrile 50% (*v/v*). Structural analysis by single crystal X-ray diffraction reveals that the asymmetric unit of the co-crystal consists of one **pentx** and one H**pca** molecule, held together by a short O-H⋯N hydrogen bond of (O···N separation of 2.700 (2) Å) involving the carboxylic acid group of protocatechuic acid and the imidazole nitrogen atom of pentoxifylline (Figure 2c).

#### 3.1.2. Salt of Clonidine and α-Lipoic Acid (Hclon^+^)(ala^−^)

**Figure 3:**
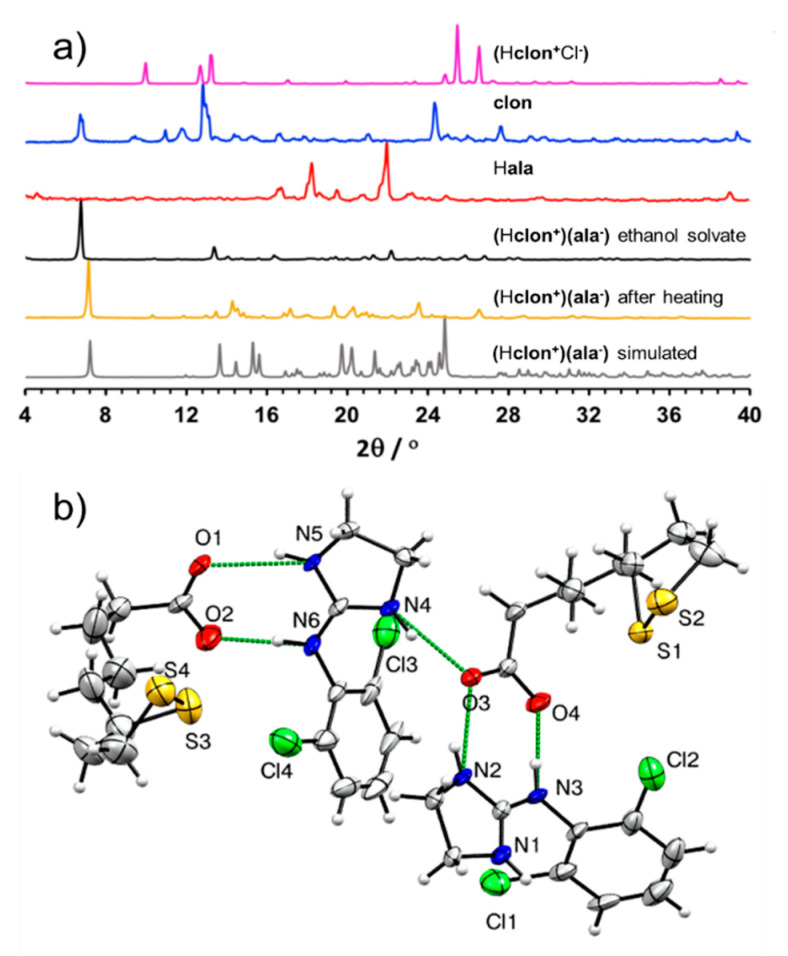
(**a**) Comparison of PXRD patterns for: (Top to bottom): (H**clon**^+^Cl^−^), solid **clon**, H**ala**, product of LAG of **clon** and H**ala** in presence of EtOH, the liquid-assisted grinding (LAG) product after heating at 95 °C for one hour and simulated for the herein reported crystal structure of (H**clon**^+^)(**ala**^−^); (**b**) view of the asymmetric unit of the (H**clon**^+^)(**ala**^−^) salt, illustrating the assembly of cations and anions via N-H···O hydrogen bonds (depicted as green dashed lines). For clarity, only non-carbon and non-hydrogen atoms have been labeled.

After being separated from its hydrochloride salt using a neutralization reaction, solid **clon** was reacted with α-lipoic acid (H**ala**) using LAG in the presence of EtOH as the liquid additive. The PXRD pattern of the material after milling was distinct from those of the corresponding starting materials, with a prominent new Bragg reflection appearing at 2*θ*-value of 6.7°, indicating the formation of a new crystalline phase (Figure 3a). Analysis of the FTIR-ATR spectrum of the product revealed a significant shift of the C=O stretch of the H**ala** carboxylic acid moiety from 1694 cm^−1^ in the pure acid to 1666 cm^−1^ in the LAG product (see Figures S9 and S10 in Supplementary Materials). The shift is consistent with the deprotonation of the carboxylic acid moiety, indicating salt formation.

Analysis of the LAG product by TGA revealed a 5% loss in sample weight, taking place in one single step between ca. 50–75 °C (see Figure S2 in Supplementary Materials). Such a well-defined step indicates that the material is a solvate containing one equivalent of EtOH per two units of (H**clon^+^**)(**ala^−^**). However, attempts to obtain the solvate salt in the form of single crystals, using diethyl ether as the solvent, yielded the non-solvated material of composition (H**clon^+^**)(**ala^−^**). Structural analysis by single crystal X-ray diffraction revealed that the asymmetric unit of (H**clon^+^**)(**ala^−^**) consists of two unique sets of **ala**^−^ anions and H**clon^+^** cations. In particular, the cations and anions are organized into pairs held together by R_2_^2^(8) hydrogen bonding motifs involving two N-H groups of each H**clon**^+^ cation and the carboxylate functionality of each **ala**^−^ anion (Figure 3b) [35]. The resulting self-assembled ion pairs are further connected into one-dimensional (1-D) chains through N-H⋯O hydrogen bonds of lengths 2.601(8) Å-2.762(8) Å.

Heating of mechanochemical LAG product at 95 °C for one hour produced a material whose PXRD pattern resembles the one simulated for the crystal structure of (H**clon^+^**)(**ala^−^**) (Figure 3a). Analysis of (H**clon^+^**)(**ala^−^**) by DSC was performed in the temperature range from −20 °C and 200 °C, which revealed a sharp endothermic event at 69 °C which was interpreted as the melting point of the salt solvate concomitant to the evaporative loss of the solvent of crystallization (see Figure S5 in Supplementary Materials). This endothermic event is followed by a rapid exothermic process at 72 °C, which was interpreted as a crystallization to a new phase which then melts in a subsequent endothermic event at 88 °C. Importantly, this behavior is different from either H**ala** or **clon**, which exhibit only sharp melting point endothermic signals at 50 °C and 142 °C, respectively.

#### 3.1.3. Salt of Linsidomine and Caffeic Acid (Hlin^+^)(cafa^−^)

**Scheme 1:**
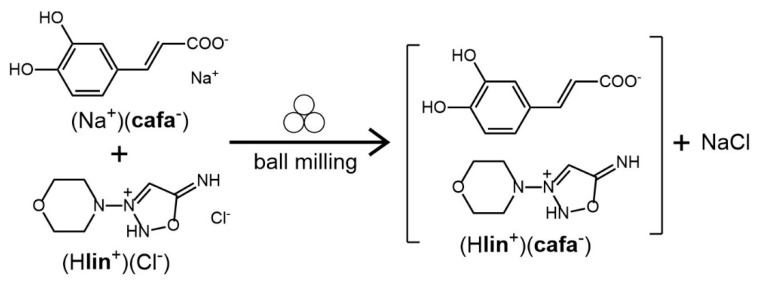
The mechanochemical double ion metathesis reaction used for the synthesis of (H**lin**^+^)(**cafa**^−^). The symbol for mechanochemical reaction conditions has been adopted from Rightmire and Hanusa [36].

As the **lin** free base is highly reactive, with the API generally available in a stable solid form as the hydrochloride salt (H**lin**^+^Cl^−^), we devised a mechanochemical double ion metathesis route for the synthesis of linsidomine salt of caffeic acid (H**lin**^+^)(**cafa**^−^), by milling of (H**lin**^+^Cl^−^) with pre-synthesized sodium salt of caffeic acid (Na^+^**cafa**^−^). The mechanochemical reaction was expected to lead to simultaneous formation of the new (H**lin**^+^)(**cafa**^−^) salt, accompanied by the byproduct NaCl (Scheme 1).

**Figure 4:**
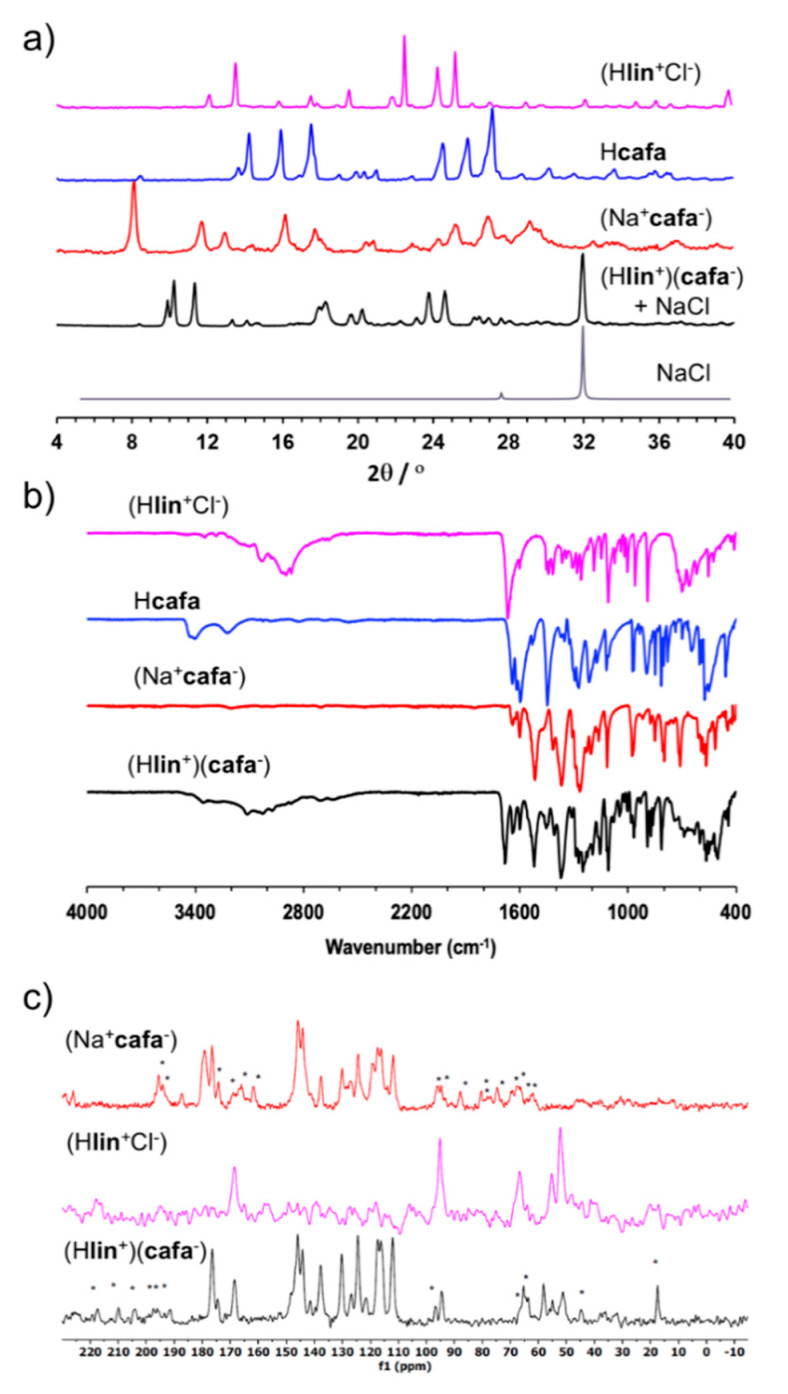
Comparison of relevant: (**a**) PXRD patterns; (**b**) FTIR-ATR spectra and (**c**) ^13^C ssNMR spectra for the synthesis of linsidomine caffeate (H**lin**^+^)(**cafa**^−^). The ssNMR side bands in (**c**) are designated by ‘*’.

Indeed, inspection of the PXRD pattern of the product immediately after milling clearly revealed X-ray reflections of sodium chloride, with the particularly characteristic one at 2*θ*-value of 31.7° (Figure 4a), providing indirect evidence for the metathesis reaction to form (H**lin**^+^)(**cafa**^−^). Moreover, the FTIR-ATR spectrum of the milled reaction mixture clearly revealed a new absorption band at 1363 cm^−1^, which is not present in the infrared spectra of either of the starting materials and is consistent with a C–O stretch of a carboxylate moiety (Figure 4b and Figure S11 in Supplementary Materials). Experiments to grow single crystals of (H**lin**^+^)(**cafa**^−^) suitable for structural characterization were unsuccessful, as the compound appeared to easily degrade upon dissolution in several different solvents. Consequently, (H**lin**^+^)(**cafa**^−^) was analyzed in more detail using DSC thermal analysis and ssNMR spectroscopy. Solid-state NMR is a standard and highly useful technique for characterizing solid materials, as it is sensitive to the chemical environment of the nuclei involved and permits recognizing the formation of new solid materials [37].

The DSC thermogram for (H**lin**^+^)(**cafa**^−^) exhibits a sharp endothermic signal at 70 °C that is consistent with melting and also confirms the absence of starting materials (see Figure S6 in Supplementary Materials). In particular, none of the starting material solids (H**lin**^+^Cl^−^), H**cafa** or (Na^+^**cafa**^−^) exhibits a sharp endothermic event that could be associated with melting: (H**lin**^+^Cl^−^) exhibits a weak endothermic event at 87 °C followed by exothermic decomposition at ca. 175 °C, the DSC thermogram for H**cafa** is featureless in the range −20 °C to 200 °C, and (Na^+^**cafa**^−^) undergoes a complex thermal transformation involving multiple endo-and exothermic events above ca. 150 °C. All these observations are consistent with the formation of (H**lin**^+^)(**cafa**^−^) as a well-defined crystalline phase, different from any of the used starting materials.

The ^13^C ssNMR CPMAS spectrum of (H**lin**^+^)(**cafa**^−^) generally resembles a combination of the solid-state spectra of the precursor salts (Na^+^**cafa**^−^) and (H**lin**^+^Cl^−^), which is consistent with the presence of both H**lin**^+^ and **cafa**^−^ in the final product (Figure 4c). However, the spectrum of (H**lin**^+^)(**cafa**^−^) also reveals minor changes in the chemical shifts of signals, which is consistent with a different crystalline environment compared to either of the starting material salts. The spectrum also reveals the appearance of additional resonances, which might suggest an increase in *Z′*, the number of moieties in the crystallographic asymmetric unit, compared to the precursor salts.

### 3.2. The Enhanced Analgesic Effects of the Nutraceutical Co-Crystal and Salts of **pentx**, **clon**, and **lin**

**Figure 5:**
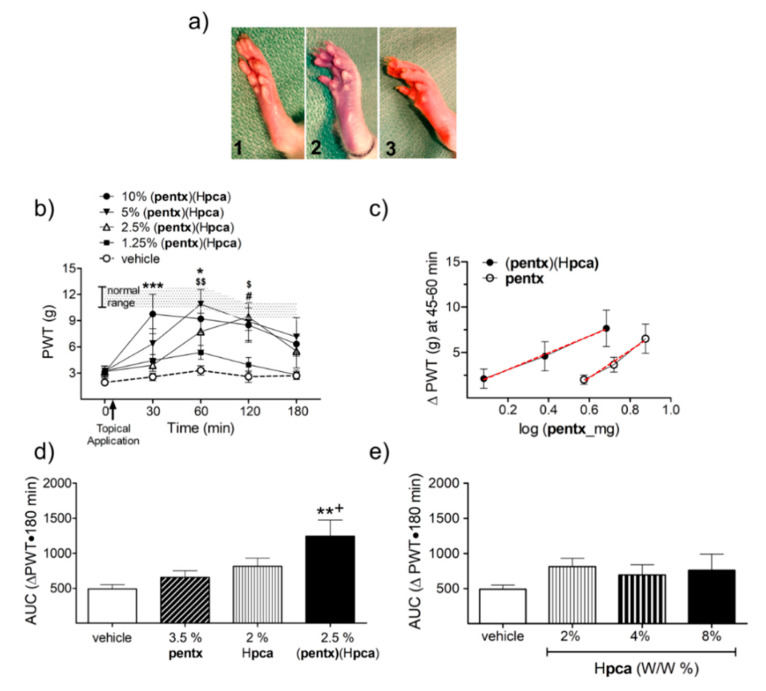
A comparison of the topical anti-allodynic effects of (**pentx**)(H**pca)** to its constituent drugs on chronic post-ischemia pain (CPIP) rats. (**a**) Appearance of a rat hind paw before (1), during (2), and 5 min (3) after application of O-ring tourniquet during preparation of CPIP rats. (**b**) time course of paw withdrawal threshold (PWT) response profile to topical application of different concentrations of (**pentx**)(H**pca)** (the shaded area represents PWT values from vehicle-treated normal animals). (* *p* < 0.05, *** *p* < 0.001 pre- versus post-drug PWT of 10% *w/w* (**pentx**)(H**pca**); **^$^** *p* < 0.05, **^$$^** *p* < 0.01 that of 5% *W/W* and **^#^** *p* < 0.05 that of 2.5% *w/w*) (*n* =8). (**c**) The anti-allodynic effect achieved by 2.5% (**pentx**)(H**pca)** exceeds the level obtained with 2 times the concentration of pentoxifylline and H**pca** contained in it; red line depicits regression line. (**d**) The (**pentx**)(H**pca)** co-crystal has greater analgesic effect than pentoxifylline as evidenced by a leftward shift in its dose–response curve, (** *p* < 0.01 compared to 3.5% **pentx**, **^+^** *p* < 0.05 compared to 2% H**pca**). (**e**). The *w/w* amounts of H**pca** contained in the anti-allodynic concentrations of (**pentx**)(H**pca)** exhibit no significant anti-allodynic effect compared to vehicle.

The synthesized co-crystals and salts were prepared in PEG as topical formulations and tested at different doses on the hind paw of CPIP rats. Occlusion of blood flow to the left hind paw with an O-ring tourniquet for 3 h followed by reperfusion renders the hind paw edematous and hyperemic for 24–48 h (Figure 5a). This subsides gradually to gives way into hypersensitivity to thermal and mechanical stimuli (allodynia) for more than three weeks after the experimentally induced ischemia reperfusion injury (IRI) [32]. The potency and efficacy of each co-crystal and salt in reducing mechanical allodynia was assessed by measuring the PWT of CPIP rats after topical application of the agents, as compared with that obtained with their corresponding constituent agents.

The topical application of the (**pentx**)(H**pca)** co-crystal produced significant anti-allodynic effects 30 to 120 min post-application at doses 2.5, 5 and 10% *w/w* (Figure 5b). A comparison of the log-dose–response curve of (**pentx**)(H**pca)** with **pentx** showed a significant leftward shift by 0.59 log units which translates into a 3.9-fold increase in potency (*p* = 0.0113) (Figure 5c). Moreover, the cumulative three h anti-allodynic effect of the lowest effective dose of (**pentx**)(H**pca)** (2.5%, constituted of 1.5% **pentx** and 1% H**pca** *w/w*) significantly exceeded the effect achieved by double the amount of its constituent drugs (3.5% **pentx** and 2% H**pca** *w/w*) (Figure 5d). Protocatechuic acid, topically applied at the different *w/w* doses did not produce significant anti-allodynic effects as compared to vehicle (Figure 5e).

**Figure 6:**
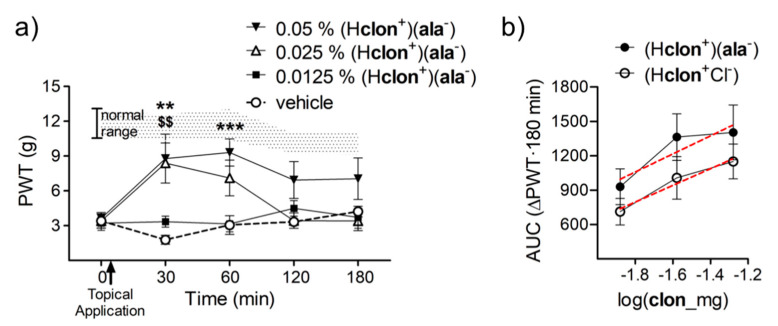
The effect of topical application of ethanol solvated (H**clon**^+^)(**ala**^−^) and how it compares to its generically available counterpart (H**clon**^+^Cl^−^). (**a**) The time course of change in PWTs after topical application of different doses of (H**clon**^+^)(**ala**^−^). The shaded area represents PWT values from vehicle-treated normal animals (** *p* < 0.01 and *** *p* < 0.001 pre-versus post-drug PWT of 0.05% *w/w* (H**clon**^+^)(**ala**^−^) and **^$$^** *p* < 0.01 that of 0.025% *w/w*) (*n* = 8). (**b**) The cumulative 3 h change in PWTs achieved by topical application of (H**clon**^+^)(**ala**^−^) as compared to (H**clon**^+^Cl^−^) shows a leftward shift in its dose–response curve implying a two-fold increase its potency (red line depicits regression line).

With topical preparation of (H**clon**^+^)(**ala**^−^) ethanol solvate, a *w/w* concentration of 0.025% brought about a significant rise in PWTs at 30 min post-application and with a dose of 0.05% the PWTs stayed elevated for twice as long (Figure 6a). The change in PWTs observed over a 3 h time course of testing after topical application of (H**clon**^+^)(**ala**^−^), as compared to (H**clon**^+^Cl^−^), shows a small but significant improvement in its anti-allodynic potency. The x-intercept of the dose–response curve for (H**clon**^+^)(**ala**^−^), as compared to (H**clon**^+^Cl^−^), shifted significantly leftward by 0.265 log units (*p* = 0.04), which converts to a nearly two-fold increase in potency (Figure 6b).

**Figure 7:**
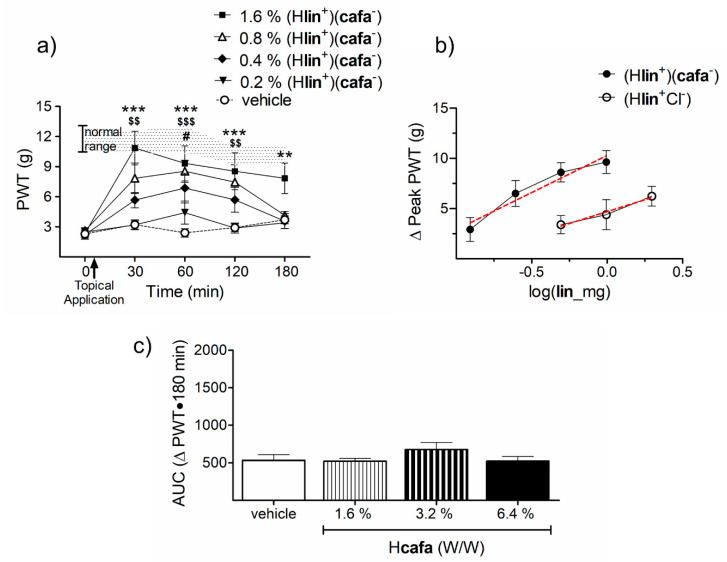
The anti-allodynic effects of topical (H**lin**^+^)(**cafa**^−^) and its dose-response comparison to (H**lin**^+^Cl^−^). (**a**) Topical (H**lin**^+^)(**cafa**^−^) significantly relieves CRPS allodynia at *w/w* doses of 1.6% and 0.8% for as long as 120 min post-application (The shaded area represents PWT values from vehicle treated normal animals.) (** *p* < 0.01, *** *p* < 0.001 pre-versus post-drug PWT of 1.6% *w/w* (H**lin**^+^)(**cafa**^−^); **^$$^** *p* < 0.01, **^$$$^** *p* < 0.001 that of 0.8% *w/w* and **^#^** *p* < 0.05 that of 0.4% *w/w* (*n* = 9)). (**b**) The dose–response curve of (H**lin**^+^)(**cafa**^−^) reaches a higher peak and is shifted leftward as compared to (H**lin**^+^Cl^−^), suggesting its greater efficacy and potency (red line depicits regression line). (**c**) Topical administration of H**cafa** on its own exhibited no anti-allodynic effects.

The *w/w* concentrations of 1.6% of (H**lin**^+^)(**cafa**^−^) significantly elevated PWTs to non-allodynic values at 30 (*p* < 0.0001), 60 (*p* < 0.0001), 120 (*p* < 0.001) and 180 (*p* < 0.01) min post-application (Figure 7a). This anti-allodynic effect is dose-dependent with each doubling of dose producing proportional rise in PWTs. A comparison of the dose–response curve of (H**lin**^+^)(**cafa**^−^) with (H**lin**^+^Cl^−^) showed the (H**lin**^+^)(**cafa**^−^) salt to have greater efficacy and potency. The maximum change in PWTs obtained after topical application of the HCl salt is (6.23 ± 0.99), but the caffeate salt brings about greater rise in PWTs (9.6 ± 1.15), despite having half the **lin** content (Figure 7b). The x-intercept of the dose–response curve for (H**lin**^+^)(**cafa**^−^) is significantly shifted to the left by 0.3966 log units as compared to (H**lin**^+^Cl^−^) (*p* = 0.0026). This shift translates to a close to 2.5-fold increase in potency (Figure 7b). Topical **cafa** produced no anti-allodynic effects on its own when administered at concentrations matching what is contained in (H**lin**^+^)(**cafa**^−^) (Figure 7c).

## 4. Discussion

The co-crystal and salt solid forms made by pairing **pentx** with H**pca**, **clon** with H**ala** and **lin** with H**cafa** are, to the best of our knowledge, among the first demonstrations of effective analgesic drug-nutraceutical co-crystals and salts. The synthesis of these new solids was achieved through mechanochemical liquid-assisted grinding. In particular, whereas the (**pentx**)(H**pca**) co-crystal was immediately obtained by liquid-assisted grinding in the presence of ethanol, the same approach yielded a solvate of the salt (H**clon**^+^)(**ala**^−^), from which the non-solvated material was obtained by thermal desolvation or re-crystallization from diethyl ether. Due to the instability of the free base **lin**, whose vasodilatory action is based in decomposition to form NO [38], the analgesic salt (H**lin**^+^)(**cafa**^−^) was prepared through a mechanochemical salt metathesis procedure involving liquid-assisted grinding of the commercially available stable solid hydrochloride form (H**lin**^+^Cl^−^) with sodium caffeate (Na^+^**cafa**^−^).

The pairing of **pentx**, **clon**, and **lin** with H**pca**, H**ala** and H**cafa** into salts and co-crystals was primarily aimed at targeting peripheral tissue hypoxia and oxidative stress seen in chronic post-ischemic pain [28,32,39]. Topical and systemic administration of agents that enhance tissue oxygenation and relieve oxidative damage have previously been shown to be analgesic in chronic pain due to IRI, peripheral neuropathy, diabetes and chemotherapeutic drug toxicity [32,39,40,41,42,43,44]. However, targeting of these mechanisms in unison has not been investigated. With the topical administration of the (**pentx**)(H**pca**) co-crystal and (H**clon**^+^)(**ala**^−^) and (H**lin**^+^)(**cafa**^−^) salts to CPIP rats suffering from mechanical allodynia, we demonstrate for the first time that enhanced analgesia can be achieved with drugs that have simultaneous anti-hypoxic and antioxidant properties. We present the contrast between the anti-allodynic effect obtained with co-crystals and salts verses the individual constituent compounds. Though the experimental validation is underway, we expect the analgesic effect obtained with the co-crystals and salts to be equivalent if not superior to what could be achieved by the mere addition of their constituents. This is mainly due to the potential for enhanced physicochemical properties acquired by the salts and co-crystals via specific supramolecular interaction between their constituent drugs. A plain mixture of these APIs, that are already in the market, lack patent rights making their investability for clinical development poor. They also require complex and costly studies of efficacy and safety for regulatory approval through assessment of their mixture in varying ratios. In contrast, the novel salts and co-crystals presented herein, are not only patentable with greater likelihood of clinical advancement but also incur a much-reduced developmental cost because of the singular dosing they allow.

In conclusion, synthesis of co-crystals and salts of compounds that impact the neural and non-neural processes involved in chronic pain is an approach that has yet to be explored. Through careful matching of drug compounds based on mechanism of action and chemical structure, the discovery of better analgesics for chronic pain can be enriched.

## 5. Patents

This work has been submitted in an international (PCT) patent application PCT/CA2019/050719 by the McGill Office of Innovation. The stakeholders in the application are T.J.C, O.A.F, A.L., G.A., T.F., C.M., and D.G.

## Supplementary Materials

The following are available online at https://www.mdpi.com/1999-4923/12/12/1144/s1. The supporting information contains thermal analysis data, selected NMR spectroscopy data for (**pentx**), (H**pca**), and **clon**, and FTIR-ATR spectra of (H**clon**^+^)(**ala**) with respective starting materials and intermediates. The crystallographic data for (**pentx**), (H**pca**), and (H**clon**^+^)(**ala**^−^) is provided in CIF format. The crystallographic data in CIF format has also been deposited to the Cambridge Crystallographic Data Centre (CCDC deposition codes 1988010 and 1988011).
