# Supplementary material for: Drug-Nutraceutical Co-Crystal and Salts for Making New and Improved Bi-Functional Analgesics
Source: Pharmaceutics. 2020 Nov 26;12(12):1144. doi: 10.3390/pharmaceutics12121144 (PMC7761172; doi:10.3390/pharmaceutics12121144)
Supplement: Supplementary file 1 [file pharmaceutics-12-01144-s001.pdf]

pharmaceutics

MDPI

Article

Supplementary Materials: Drug-Nutraceutical Co-Crystal and Salts for Making New and Improved Bi-Functional Analgesics

Oli Abate Fulas, André Laferrière, Ghada Ayoub, Dayaker Gandrath, Cristina Mottillo, Hatem M. Titi, Robin S. Stein, Tomislav Friščić and Terence J. Coderre

TABLE OF CONTENTS

1. SUPPLEMENTARY METHODS 3

1.1 [15]N Solid State Nuclear Magnetic Resonance Spectroscopy (ssNMR) 3

2. SUPPLEMENTARY DATA 4

2.1 Thermogravimetric Analysis 4

2.1.1 Thermogravimetric analysis of (**pentx**)(H**pca)** 4

2.1.2 Thermogravimetric analysis of ethanol solvated (H**clon**$^{+}$)(**ala**$^{-}$) 5

2.1.3 Thermogravimetric analysis of **(Hlin**$^{+}$)(**cafa**$^{-}$**)** 6

2.2 Differential scanning calorimetry 7

2.2.1 Differential scanning calorimetry of (**pentx**)(H**pca**) 7

2.2.2 Differential scanning calorimetry of ethanol solvated (H**clon**$^{+}$)(**ala**$^{-}$) 7

2.2.3 Differential scanning calorimetry of (Hl**in**$^{+}$)(**cafa**$^{-}$) 8

2.3 [15]N Solid State Nuclear Magnetic Resonance Spectroscopy (ssNMR) of (**pentx**)(H**pca**) 8

2.4 [1]H Nuclear Magnetic Resonance Spectroscopy (NMR) of **clon** 9

2.5 FTIR-ATR spectra 9

2.6 Crystal data and refinement details 101

1. Supplementary Methods

*[15]N Solid State Nuclear Magnetic Resonance Spectroscopy (ssNMR)***.**

^{15}N ssNMR on was performed using a Varian 400 MHz VNMRS wide bore spectrometer operating at a ^{15}N frequency of 40.51 MHz. ^{15}N CPMAS spectra were acquired using a 7.5 mm double-resonance probe. Both **pentx** and the (**pentx**)(H**pca**)co-crystal spectra were acquired under spinning at 5 kHz using a recycle delay of 20 s and contact time of 4 ms and 2560 scans were acquired of **pentx** and 1024 scans were acquired of the co-crystal. ^{15}N spectra were referenced using glycine at 33.4 ppm.

2. Supplementary Data

2.1. Thermogravimetric Analysis

2.1.1. Thermogravimetric analysis of (pentx)(Hpca)

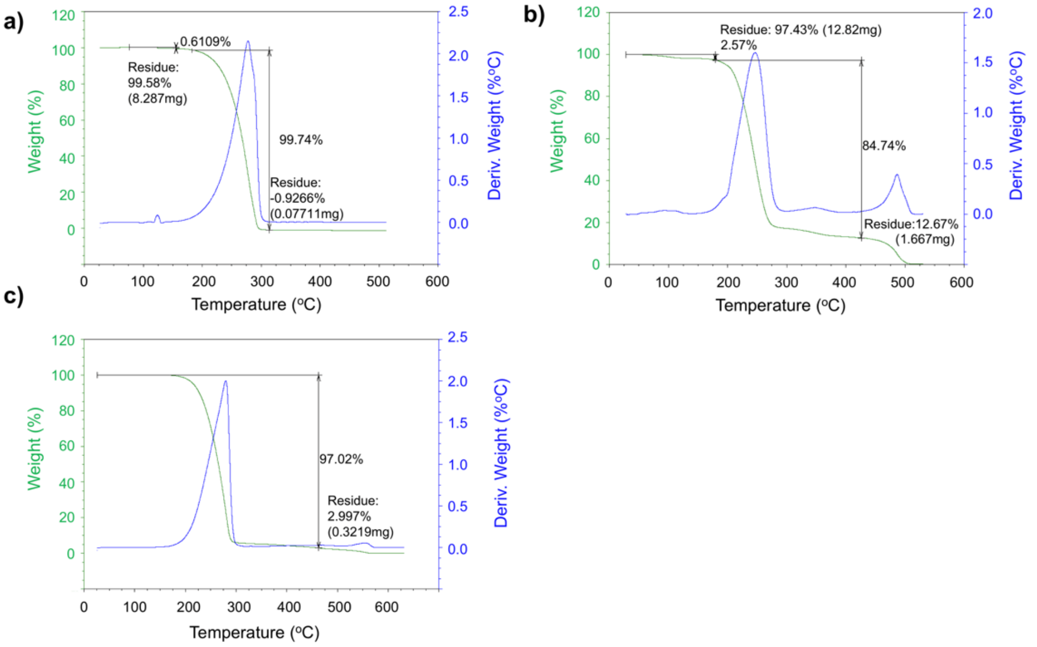

Figure S1. TGA thermogram for **pentx** (a), H**pca** (b) and **(pentx)(**H**pca)** (c).

2.1.2. Thermogravimetric analysis of ethanol solvated (Hclon$^+$)(ala$^-$)

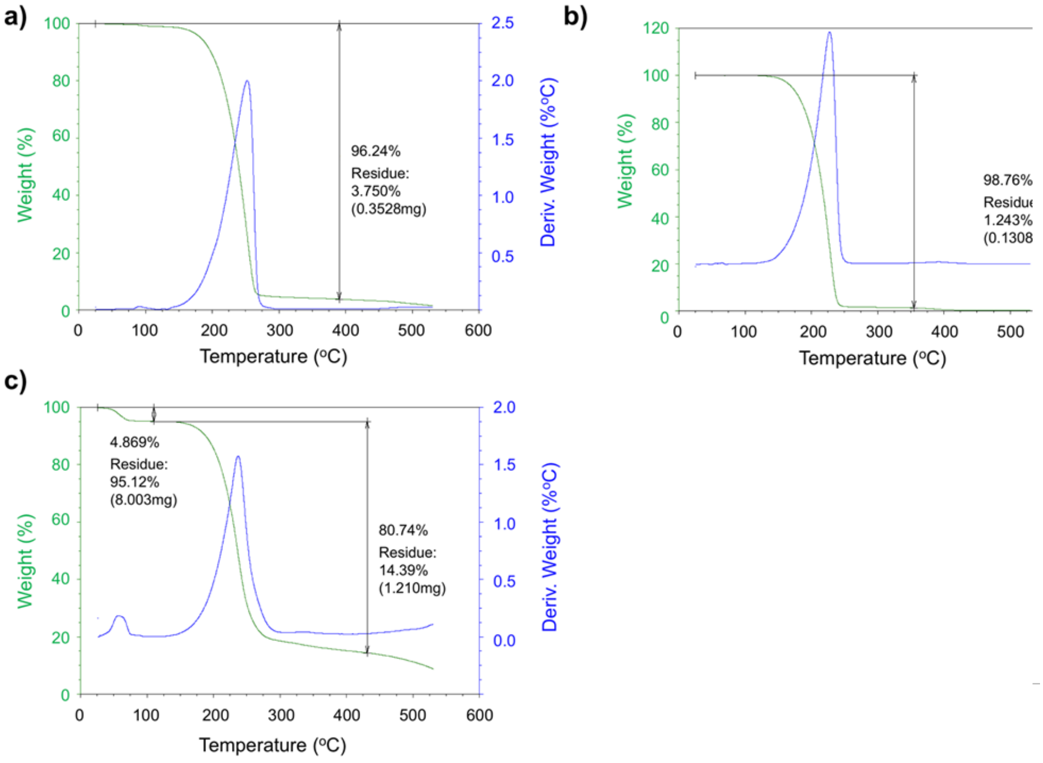

Figure S2. TGA thermogram for **clon** (a), H**ala** (b) and ethanol solvated (H**clon**$^+$)(**ala**$^-$) (c).

2.1.3. Thermogravimetric analysis of (Hlin⁺)(cafa⁻)

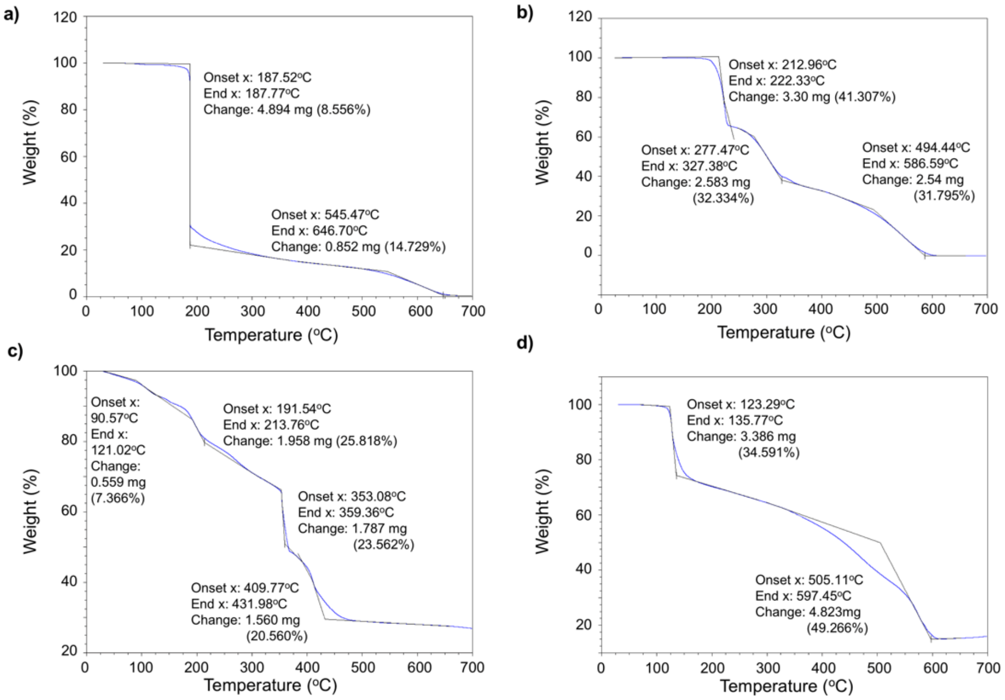

Figure S3. TGA thermogram for **(Hlin⁺Cl⁻)** (a), H**cafa** (b) (Na⁺**cafa**⁻) (c) and **(Hlin⁺)(cafa⁻)** (d).

2.2. *Differential scanning calorimetry*

2.2.1. Differential scanning calorimetry of (pentx)(Hpca)

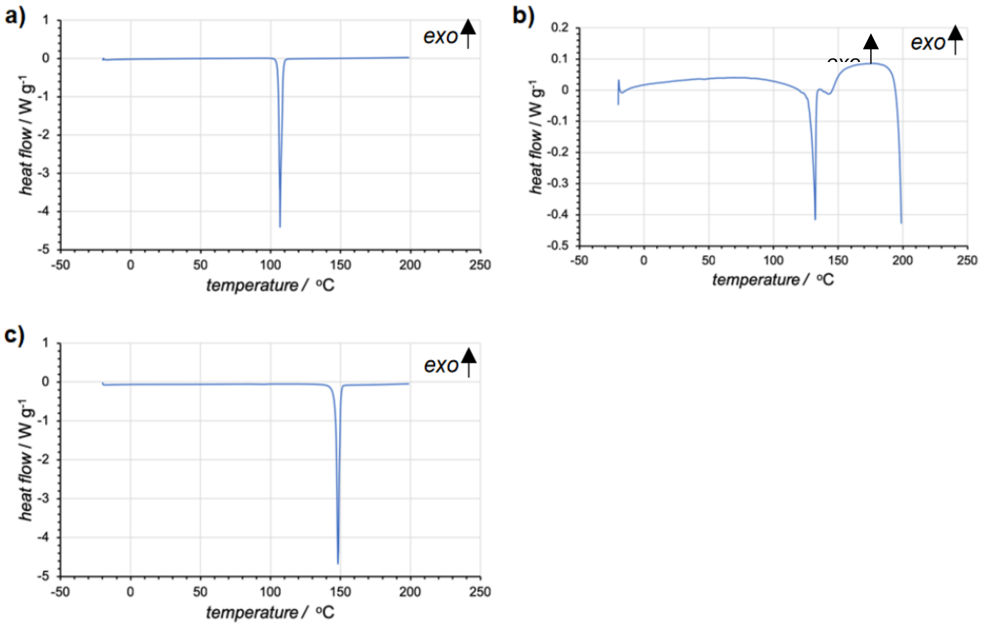

Figure S4. DSC thermogram for **pentx** (a), H**pca** (b) and **(pentx)(**H**pca)** (c).

2.2.2. Differential scanning calorimetry of ethanol solvated (Hclon⁺)(ala⁻)

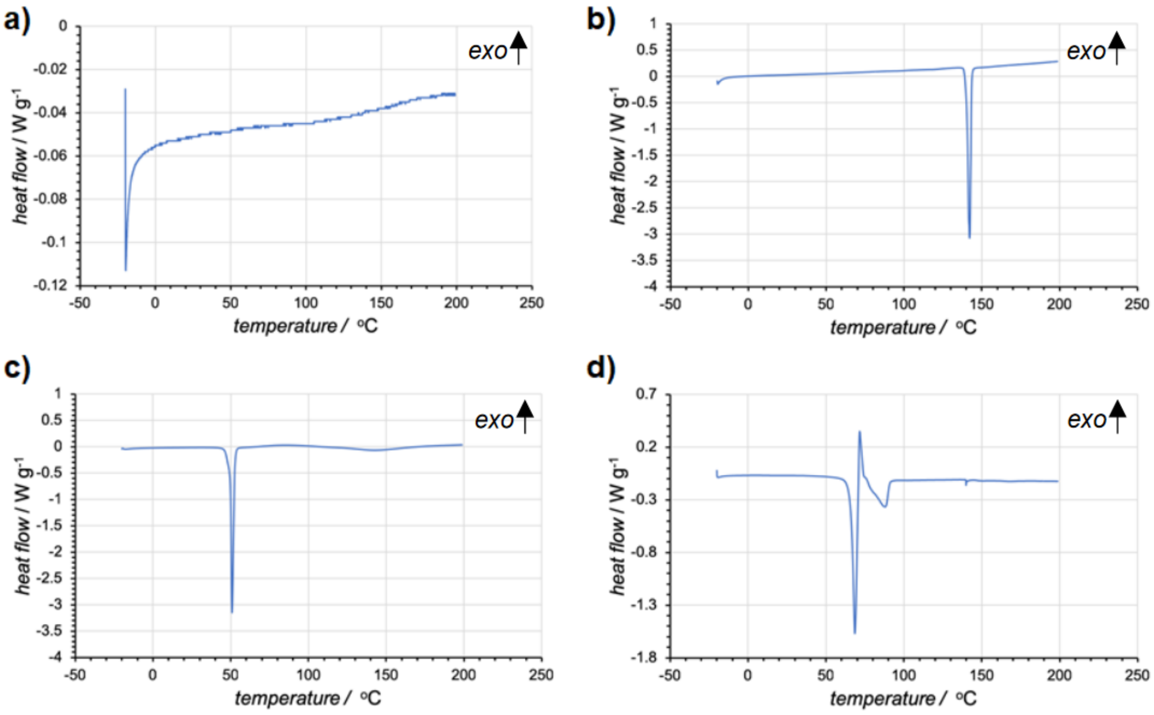

Figure S5. DSC thermogram for **(**H**clon**⁺Cl⁻**)** (a), **clon** (b), H**ala** (c) and ethanol-solvated **(**H**clon**⁺)(**ala**⁻**)** (d).

2.2.3. Differential scanning calorimetry of (Hlin⁺)(cafa⁻)

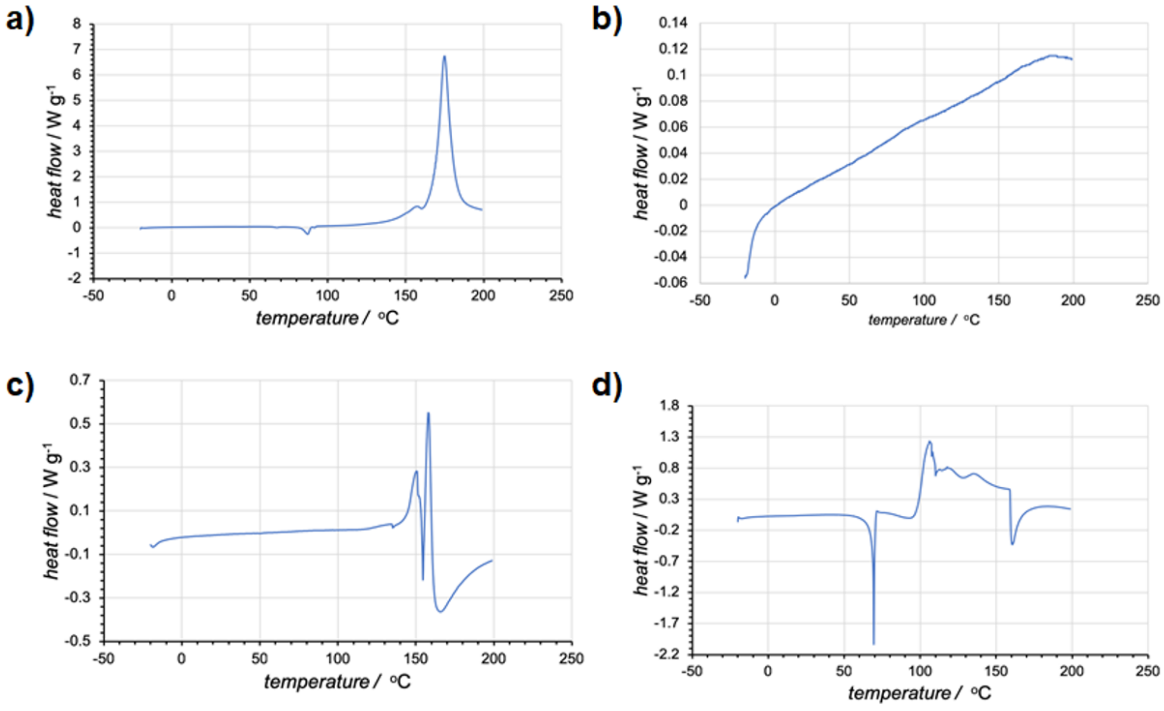

Figure S6. DSC thermogram of **(**H**lin**⁺Cl⁻**)** (a), H**cafa** (b), **(**Na⁺**cafa**⁻**)** (c) and **(**H**lin**⁺**)(cafa**⁻**)** (d).

2.3 [15]N Solid State Nuclear Magnetic Resonance Spectroscopy (ssNMR) of (pentx)(Hpca)

The largest chemical shift change is at the C=N site of **pentx** (217.7 ppm in the component and 230.3 ppm in the complex), which is the site that interacts most strongly with the H**pca**, congruent to what is observed in the crystal structure.

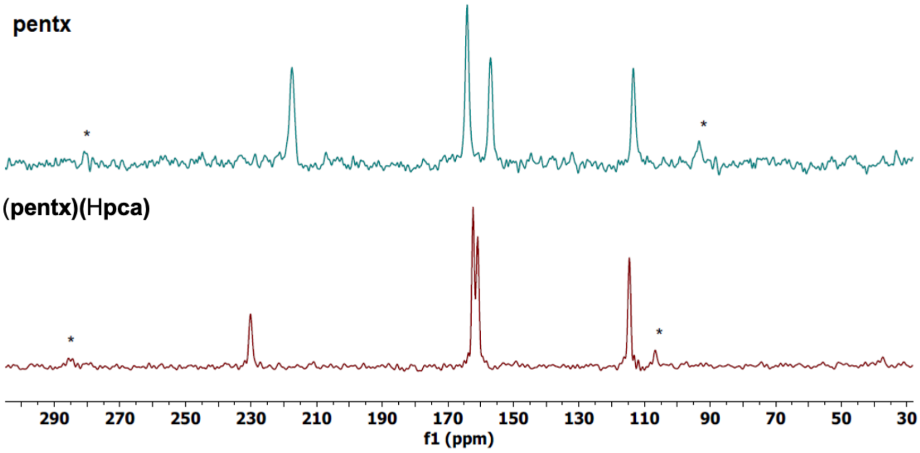

Figure S7. ^{15}N ssNMR of **pentx** and **(pentx)(Hpca).** Spinning sidebands are indicated by *.

2.4 [1]H Nuclear Magnetic Resonance Spectroscopy (NMR) of clon

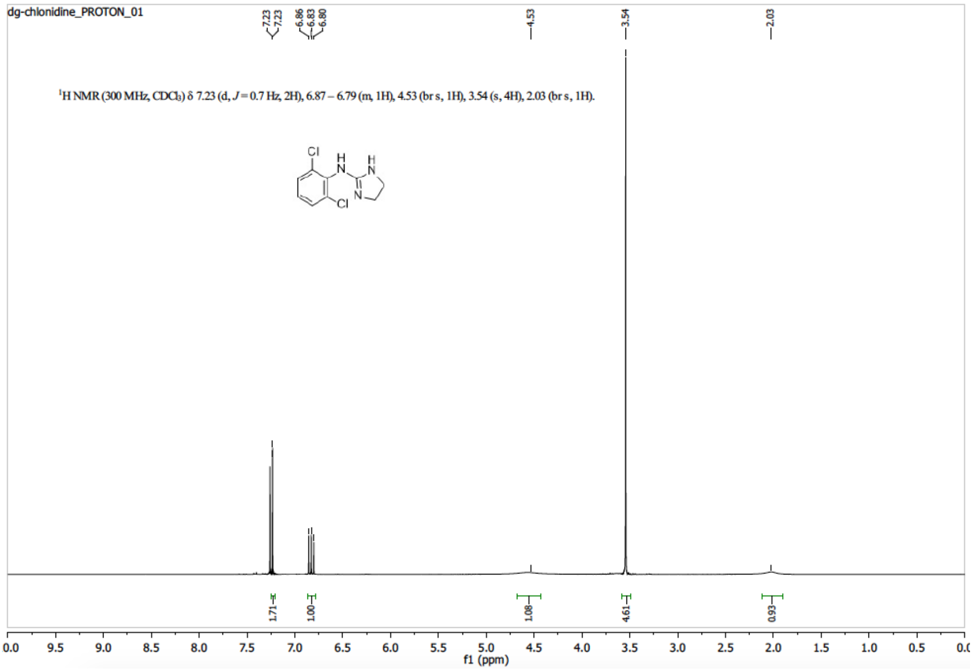

Figure S8. ^{1}H NMR spectrum (300 MHz) of **clon** after reaction with KOH.

2.5. FTIR-ATR spectra

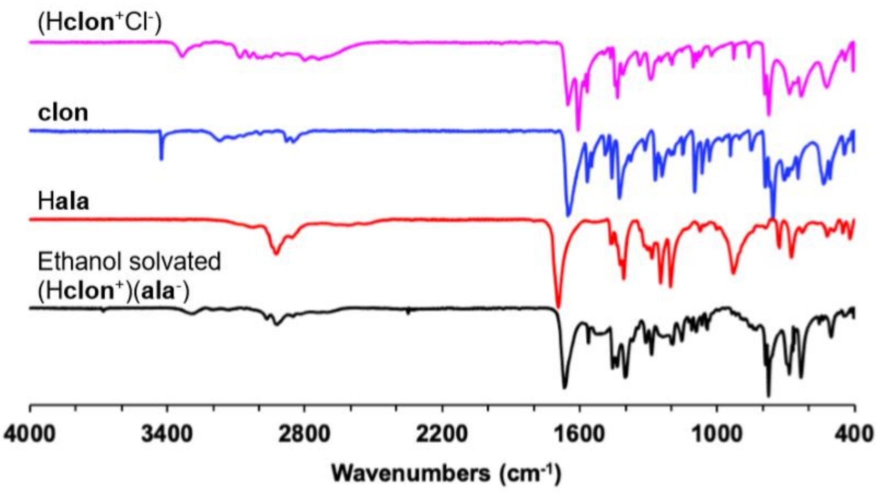

Figure S9. FTIR-ATR spectra for (H**clon**$^+$Cl$^-$), **clon**, H**ala** and ethanol solvated (H**clon**$^+$)(**ala**$^-$).

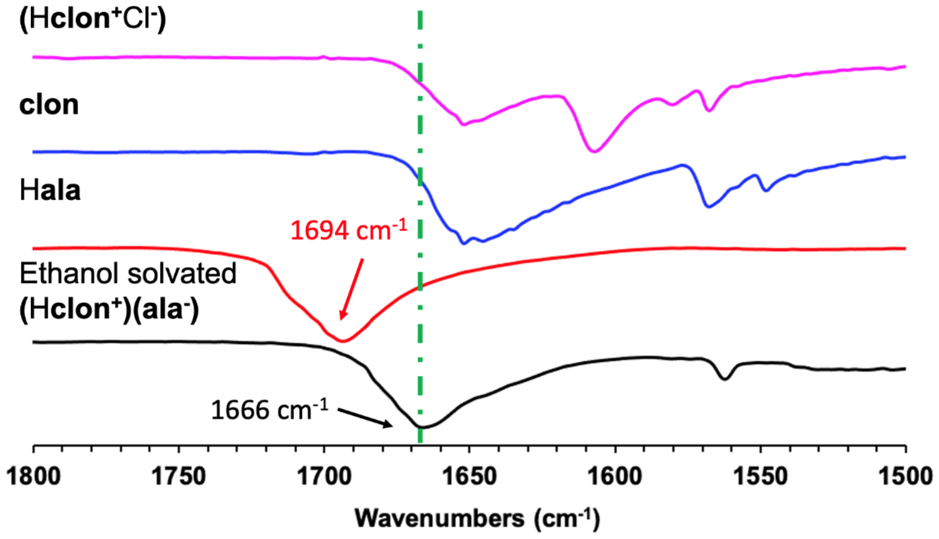

Figure S10. Selected region of FTIR-ATR spectra for (H**clon**$^+$Cl$^-$), **clon**, H**ala** and ethanol solvated (H**clon**$^+$)(**ala**$^-$).

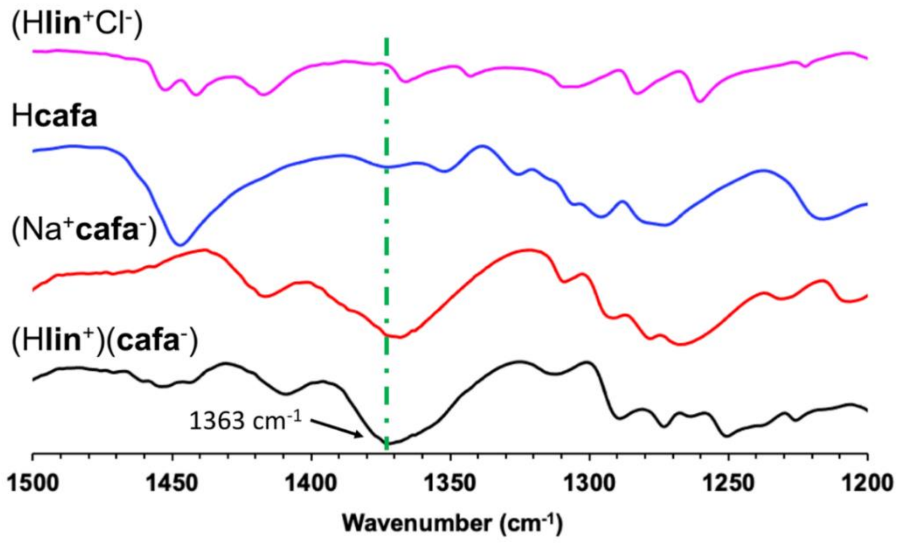

Figure S11. Selected region of FTIR-ATR spectra for (H**lin**$^+$Cl$^-$), H**cafa**, (Na$^+$**cafa**$^-$) and (H**lin**$^+$)(**cafa**$^-$).

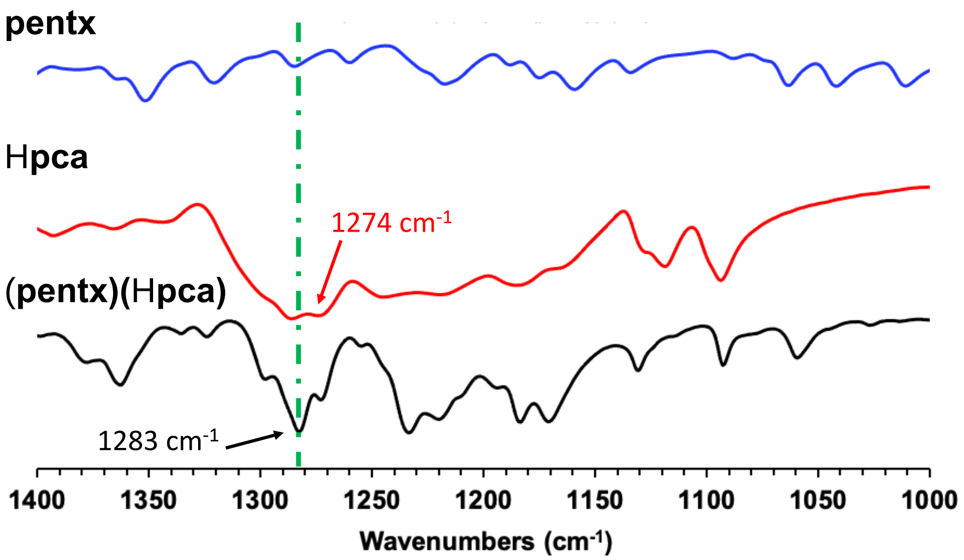

Figure S12. Selected region of FTIR-ATR spectra for (**pentx**), H**pca** and (**pentx**)(H**pca**).

2.6. Crystal data and refinement details

2.6.1. Crystal packing for (pentx)(Hpca)

The crystal packing of (**pentx**)(H**pca**) reveals a range of hydrogen bonding interactions. The carboxylic acid group of H**pca** forms a hydrogen bond to the nitrogen atom N12 of **pentx**, while the hydroxyl moieties of H**pca** form O-H···$O_{carbonyl}$ interactions with the carbonyl groups of **pentx**. These hydrogen bonds give rise to a ladder-like one-dimensional (1D) chain along the crystallographic *b*-axis, as shown in Figure S13. The 1D hydrogen-bonded chains are connected through C-H···$O_{carbonyl}$ hydrogen-bonding contacts and, additionally, the molecules also form π···π stacking motifs along the crystallographic *a*-axis, with a repeat distance of 3.30 Å.

The starting material H**pca** (CSD code WUYNUA) crystallizes in triclinic *P*-1 space group and exhibits three different independent molecules in the asymmetric unit. Two crystallographically distinct sets of H**pca** molecules form 2-dimensional (2D) sheets through p···p stacks and hydrogen-bonding motifs between the hydroxyl and carboxylic acid functionalities. Each of these sheets is hydrogen-bonded to a different type of 2D layers, which is formed by the third crystallographically

distinct set of H**pca** molecules (Figure S14a). The molecules in these two alternating sets of 2D layers are mutually perpendicular.

The starting material **pentx** is known to form two polymorphs [CSD codes JAKGEH (triclinic *P*-1) and JAKGEH01 (monoclinic $P2_1/c$)], in which the alkyl chain of the molecule adopts different orientations. Both structures display weak C-H···O hydrogen bonding and p···p stacking. The crystal packing in the structures with CSD codes JAKGEH and JAKGEH01 reveals hydrophobic regions propagating along the crystallographic *a*-axis and in the (110) set of crystallographic planes, respectively.

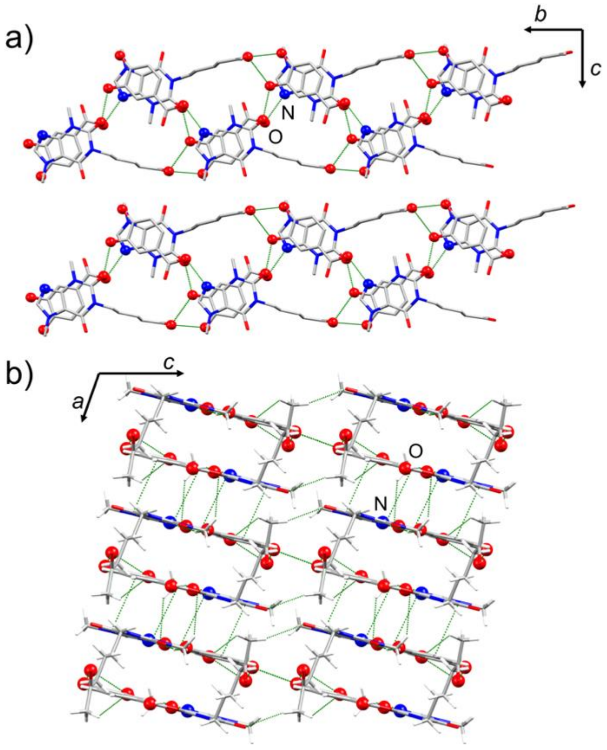

Figure S13. Crystal packing for the cocrystal (**pentx**)(H**pca**) viewed a long: a) the crystallographic *a*-axis and b) along the crystallographic *b*-axis (b). Hydrogen atoms in a) have been omitted for clarity.

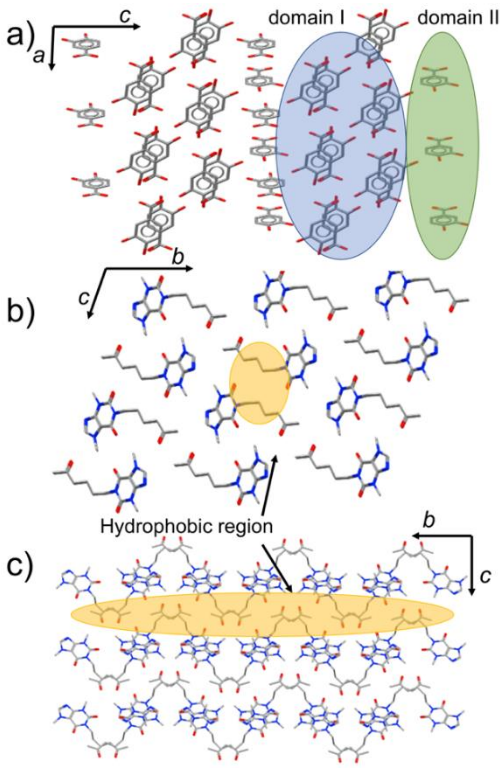

Figure S14. Crystal structures of solid H**pca** (CSD code WUYNUA) and the two polymorphs of solid pent (CSD codes JAKGEH and JAKGEH01): a) layers in the structure of H**pca** viewed along the crystallographic *b*-axis; b) structure with the CSD code JAKGEH viewed along the crystallographic *a*-axis; and c) structure with the CSD code JAKGEH01 viewed along the crystallographic *a*-axis.

2.6.2. Crystal packing for (Hclon$^+$)(ala$^-$)

The crystal packing of (H**clon**$^+$)(**ala**$^-$) can be described through two domains of H**clon**$^+$ and **ala**$^-$ molecular ions stacked along the crystallographic *b*-axis. These ions are held together by short hydrogen bonds involving the carboxylate and 4,5-dihydroimidazolium functionalities to form 1D hydrogen-bonded sheets (Figure S15). In addition, the **ala**$^-$ ions are involved in weaker C-H···O hydrogen bonds and van der Waals interactions between these 1D hydrogen-bonded polymers. In comparison, the structure of solid **clon** (CSD code MUFBUK) exhibits two crystallographically non-equivalent molecules in the asymmetric unit and adopts the monoclinic space group $P2_1/c$ (Figure S16a and b). In the structure, the **clon** molecules are engaged through a series of hydrogen bonding interactions between the 4,5-dihydroimidazole units (hydrogen bond distance of 2.98 Å) to form dimers. These hydrogen bonded dimers further interact through Type I Cl···Cl interactions (slightly shorter than the sum of vdW radii, 3.40 Å) to form 1D supramolecular chains. The crystal structure of pure solid H**ala** (CSD code THOCAR01) exhibits one molecule in the asymmetric unit, and adopts the monoclinic $P2_1$/c space group. The molecules in this structure are interacting through hydrogen bonds between the carboxylic acid moieties to form $R_2^2(8)$ dimer motifs, as well as through C-H···S hydrogen bonds (Figure S16c and d).

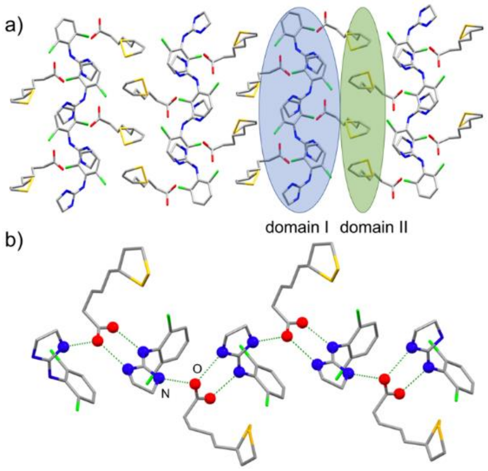

Figure S15. Crystal packing for the cocrystal (H**clon**$^{+}$)(**ala**$^{-}$) through *b*-axis (a) and illustration of the 1D hydrogen bonding chains (b). Note that hydrogen atoms were omitted for clarity.

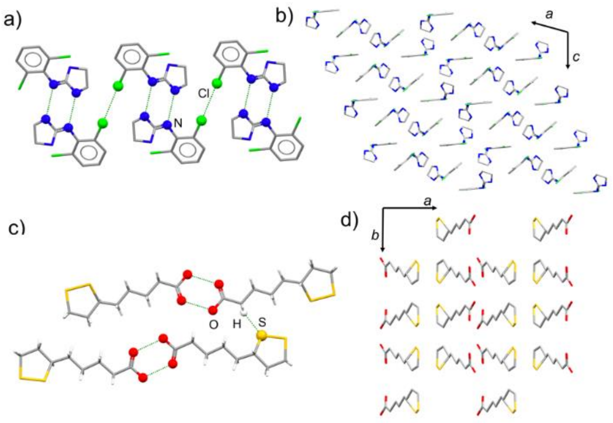

Figure S16. Crystal structure of **clon** (CSD code MUFBUK): a) hydrogen bonding and Cl···Cl interactions and b) view of the crystal packing along the crystallographic *b*-axis. Crystal structure of H**ala** (CSD code THOCAR01): c) hydrogen bonding motifs and d) crystal packing viewed along the crystallographic *c*-axis. Hydrogen atoms in some figures were omitted for clarity.

**Table S1. Crystal data and structure refinement for (pentx)(Hpca) and (Hclon⁺)(ala⁻)**		
**Identification code**	**(pentx)(Hpca)**	**$(Hclon^+)(ala^-)$**
Empirical formula	$C_{20}H_{24}N_4O_7$	$C_{17}H_{23}Cl_2N_3O_2S_2$
Formula weight	432.43	436.40
Temperature/K	298(2)	273(2)
Crystal system	monoclinic	monoclinic
Space group	$P2_1$	$P2_1$
a/Å	6.8500(4)	9.5272(7)
b/Å	12.5837(6)	8.8398(6)
c/Å	12.1661(6)	24.4933(17)
α/°	90	90
β/°	100.225(4)	92.065(4)
γ/°	90	90
Volume/Å^3	1032.04(9)	2061.5(3)
Z	2	4
ρ_{calc}g/cm^3	1.392	1.406
μ/mm^{-1}	0.898	4.868
F(000)	456.0	912.0
Radiation	CuKα (λ = 1.54178)	CuKα (λ = 1.54178)
2θ range for data collection/°	7.384 to 139.312	7.222 to 120.364
Reflections collected	3580	19058
Independent reflections	3580 [R_{int} = 0.1808, R_{sigma} = 0.0478]	6053 [R_{int} = 0.0706, R_{sigma} = 0.0808]
Data/restraints/parameters	3580/1/287	6053/374/470
Goodness-of-fit on F^2	1.054	1.051
Final R indexes [I>=2σ (I)]	R_1 = 0.0476, wR_2 = 0.1101	R_1 = 0.0623, wR_2 = 0.1468
Final R indexes [all data]	R_1 = 0.0656, wR_2 = 0.1210	R_1 = 0.0900, wR_2 = 0.1580
Largest diff. peak/hole / e Å^{-3}	0.22/-0.24	0.61/-0.35
Flack parameter	0.61(19)	0.04(3)

Table S2. Fractional Atomic Coordinates (×10^4) and Equivalent Isotropic Displacement Parameters (Å^2×10^3) for (pentx)(Hpca). U_{eq} is defined as 1/3 of of the trace of the orthogonalised U_{IJ} tensor

Atom	*x*	*y*	*z*	U(eq)
O1	2612(8)	7192(3)	5607(3)	53.7(12)
C2	2544(9)	8213(5)	5304(5)	41.6(15)
O3	2171(7)	8493(3)	4336(3)	60.3(12)
C4	2975(8)	8946(4)	6264(5)	34.7(14)
C5	2972(9)	10035(4)	6040(5)	36.0(14)
C6	3388(8)	10745(4)	6912(5)	37.2(13)
C7	3842(8)	10393(5)	8008(4)	37.6(14)
C8	3796(9)	9312(4)	8224(5)	40.3(15)
C9	3360(8)	8593(5)	7355(5)	39.0(13)
O10	3415(8)	11822(3)	6765(3)	52.7(11)
O11	4294(7)	11072(3)	8886(3)	53.1(12)
N12	1994(7)	5931(4)	3783(4)	40.6(12)
C13	1620(9)	6388(5)	2789(5)	43.7(15)
N14	1217(7)	5706(4)	1942(4)	39.0(12)
C15	1341(8)	4694(5)	2427(4)	34.8(13)
C16	1837(8)	4876(4)	3544(4)	33.5(13)
C17	737(10)	5965(6)	751(5)	57.3(18)
N18	2132(7)	4069(3)	4300(4)	37.6(12)
C19	1976(8)	3042(5)	3939(5)	37.3(14)
N20	1398(6)	2875(3)	2796(4)	36.2(11)
C21	1088(8)	3665(5)	1965(5)	36.9(13)
C22	2690(10)	4285(5)	5500(4)	51.3(17)
O23	2308(7)	2287(3)	4574(3)	51.8(12)
O24	638(6)	3436(3)	974(3)	54.5(12)
C25	1248(9)	1753(4)	2426(5)	44.8(16)
C26	3224(8)	1391(5)	2170(5)	47.8(15)
C27	3350(8)	219(4)	1893(5)	44.6(15)
C28	5427(10)	-19(4)	1650(6)	48.4(17)
C29	5861(11)	-1158(5)	1459(5)	51.7(18)
O30	4563(7)	-1832(3)	1314(4)	64.0(13)
C31	8013(10)	-1441(6)	1456(6)	69(2)

Table S3. Anisotropic Displacement Parameters (Å^2×10^3) for (pentx)(Hpca). The Anisotropic displacement factor exponent takes the form: $-2\pi^2[h^2a^{*2}U_{11}+2hka^*b^*U_{12}+\ldots]$

Atom	U_{11}	U_{22}	U_{33}	U_{23}	U_{13}	U_{12}
O1	77(3)	34(2)	47(2)	-7(2)	3(3)	3(2)
C2	45(3)	30(3)	51(4)	-7(3)	13(3)	-3(3)
O3	94(3)	45(3)	41(3)	-5(2)	11(3)	-2(3)
C4	38(3)	29(3)	38(3)	-3(2)	9(3)	0(3)
C5	40(3)	36(3)	32(3)	0(2)	6(3)	2(3)
C6	45(3)	25(3)	41(3)	0(3)	8(3)	-1(3)
C7	43(3)	39(3)	33(3)	-5(3)	9(3)	-1(3)
C8	48(4)	33(3)	38(3)	6(3)	3(3)	-2(3)
C9	40(3)	26(3)	49(4)	1(3)	3(3)	1(3)
O10	84(3)	31(3)	41(2)	0.8(19)	7(3)	-1(2)
O11	81(3)	37(2)	40(3)	-1.5(19)	7(2)	-10(2)
N12	43(3)	32(3)	45(3)	-4(2)	4(2)	3(2)
C13	49(4)	32(3)	49(4)	-2(3)	7(3)	8(3)
N14	46(3)	39(3)	31(3)	4(2)	5(2)	9(3)
C15	37(3)	35(3)	32(3)	3(3)	6(2)	4(3)
C16	35(3)	30(3)	35(3)	-2(3)	4(3)	4(3)
C17	76(5)	61(5)	34(3)	12(3)	7(3)	12(4)
N18	46(3)	33(3)	32(3)	-3(2)	3(2)	0(2)
C19	42(3)	33(3)	37(3)	-8(3)	7(3)	3(3)
N20	42(3)	28(3)	38(3)	-4(2)	6(2)	1(2)
C21	40(3)	33(3)	37(3)	-6(3)	5(3)	5(3)
C22	74(5)	49(4)	28(3)	-7(3)	2(3)	0(4)
O23	79(3)	34(2)	41(3)	3(2)	6(2)	1(2)
O24	76(3)	50(3)	34(2)	-9(2)	4(2)	7(2)
C25	58(4)	30(3)	45(4)	-10(3)	5(3)	0(3)
C26	47(3)	39(3)	58(4)	-8(3)	11(3)	0(3)
C27	54(4)	32(3)	45(4)	0(3)	2(3)	8(3)
C28	64(4)	43(4)	40(4)	4(3)	14(3)	12(3)
C29	77(5)	44(4)	36(4)	9(3)	16(3)	23(4)
O30	73(3)	39(3)	74(3)	-5(2)	-1(3)	15(3)
C31	75(5)	60(5)	81(5)	16(4)	40(4)	22(4)

Table S4. Bond Lengths for (pentx)(Hpca)					
**Atom**	**Atom**	**Length/Å**	**Atom**	**Atom**	**Length/Å**
O1	C2	1.335(7)	C15	C16	1.360(7)
C2	O3	1.213(6)	C15	C21	1.410(8)
C2	C4	1.476(7)	C16	N18	1.361(6)
C4	C9	1.380(7)	N18	C19	1.363(7)
C4	C5	1.398(7)	N18	C22	1.467(7)
C5	C6	1.377(7)	C19	O23	1.220(7)
C6	O10	1.368(6)	C19	N20	1.393(7)
C6	C7	1.387(7)	N20	C21	1.407(7)
C7	O11	1.360(6)	N20	C25	1.480(7)
C7	C8	1.388(8)	C21	O24	1.225(6)
C8	C9	1.383(8)	C25	C26	1.512(8)
N12	C13	1.322(7)	C26	C27	1.519(7)
N12	C16	1.359(7)	C27	C28	1.533(8)
C13	N14	1.331(7)	C28	C29	1.490(8)
N14	C15	1.400(7)	C29	O30	1.219(8)
N14	C17	1.465(7)	C29	C31	1.517(9)

Table S5. Bond Angles for (pentx)(Hpca)							
**Atom**	**Atom**	**Atom**	**Angle/°**	**Atom**	**Atom**	**Atom**	**Angle/°**
O3	C2	O1	122.6(5)	N12	C16	C15	112.0(5)
O3	C2	C4	124.4(5)	N12	C16	N18	126.0(5)
O1	C2	C4	113.0(5)	C15	C16	N18	122.1(5)
C9	C4	C5	119.7(5)	C16	N18	C19	119.7(4)
C9	C4	C2	122.5(5)	C16	N18	C22	121.1(5)
C5	C4	C2	117.8(5)	C19	N18	C22	119.2(5)
C6	C5	C4	119.6(5)	O23	C19	N18	122.6(5)
O10	C6	C5	123.2(5)	O23	C19	N20	120.2(5)
O10	C6	C7	115.9(5)	N18	C19	N20	117.2(5)
C5	C6	C7	120.9(5)	C19	N20	C21	126.2(5)
O11	C7	C6	122.4(5)	C19	N20	C25	116.1(5)
O11	C7	C8	118.5(5)	C21	N20	C25	117.6(5)
C6	C7	C8	119.1(5)	O24	C21	N20	121.4(5)
C9	C8	C7	120.4(5)	O24	C21	C15	126.9(6)
C4	C9	C8	120.3(6)	N20	C21	C15	111.7(5)
C13	N12	C16	103.5(5)	N20	C25	C26	109.5(4)
N12	C13	N14	114.0(5)	C25	C26	C27	115.2(5)
C13	N14	C15	105.7(5)	C26	C27	C28	108.9(5)
C13	N14	C17	127.0(5)	C29	C28	C27	115.9(5)
C15	N14	C17	127.3(5)	O30	C29	C28	122.1(6)
C16	C15	N14	104.7(5)	O30	C29	C31	121.4(6)
C16	C15	C21	123.0(5)	C28	C29	C31	116.6(7)
N14	C15	C21	132.3(5)				

Table S6. Hydrogen Bonds for (pentx)(Hpca)

D	H	A	d(D-H)/Å	d(H-A)/Å	d(D-A)/Å	D-H-A/°
O1	H1	N12	0.82	1.92	2.700(6)	158.9
O10	H10	O23[1]	0.82	1.88	2.703(6)	179.6
O11	H11	O10	0.82	2.29	2.714(5)	113.1
O11	H11	O30[2]	0.82	2.00	2.773(5)	156.6
C13	H13	O3	0.93	2.62	3.233(7)	123.9
C31	H31B	O24[3]	0.96	2.43	3.254(8)	143.6
C31	H31C	O10[4]	0.96	2.63	3.342(8)	131.7

Table S7. Hydrogen Atom Coordinates (Å×10^4) and Isotropic Displacement Parameters (Å^2×10^3) for (pentx)(Hpca)

Atom	*x*	*y*	*z*	U(eq)
H1	2736.16	6819.69	5070.61	81
H5	2692.03	10278.86	5307.73	43
H8	4060.56	9068.79	8957.58	48
H9	3325.77	7869.77	7507.13	47
H10	3073.23	11962.17	6100.12	79
H11	4411.53	11676.31	8653.49	80
H13	1636.1	7120.48	2689.91	52
H17A	1109.46	6686.82	637.78	86
H17B	-663.03	5881.01	494.33	86
H17C	1448.52	5496.8	339.93	86
H22A	2818.78	5038.13	5618.39	77
H22B	3932.61	3945.76	5783.39	77
H22C	1685.01	4013.29	5882.02	77
H25A	232.67	1684.26	1764.27	54
H25B	876.66	1310.85	3008.72	54
H26A	4244.53	1550.14	2809.55	57
H26B	3514.24	1802.61	1544.27	57
H27A	2356.56	44.42	1246.31	54
H27B	3101.21	-208.62	2517.43	54
H28A	5606.95	382.3	995.03	58
H28B	6396.59	237.45	2273.1	58
H31A	8800.9	-805.69	1535.5	103
H31B	8120.45	-1783.83	764.16	103
H31C	8478.85	-1913.02	2066.12	103

Table S8. Fractional Atomic Coordinates (×10^4) and Equivalent Isotropic Displacement Parameters (Å^2×10^3) for (Hclon$^+$)(ala$^-$). U$_{eq}$ is defined as 1/3 of of the trace of the orthogonalised U$_{IJ}$ tensor.

Atom	*x*	*y*	*z*	U(eq)
Cl1	8745(2)	4303(3)	7024.4(9)	54.4(6)
Cl2	7086(3)	1003(3)	8738.0(9)	57.9(7)
Cl3	3851(2)	9278(3)	7974.7(10)	57.7(7)
Cl4	1855(3)	6062(3)	6248.0(10)	64.4(7)
S1	2786(2)	293(3)	9442.4(8)	42.3(6)
S2	1336(3)	-1262(3)	9690.9(10)	53.1(7)
S3	-1901(3)	5196(4)	5600.5(11)	72.2(8)
S4	-3740(3)	4111(3)	5379.8(11)	69.6(8)
O1	-1474(5)	10009(7)	6878(2)	38.0(14)
O2	629(6)	10160(8)	6529(2)	56.5(18)
O3	3731(5)	5116(7)	8111.4(19)	32.0(13)
O4	5859(5)	5087(7)	8515(2)	41.2(14)
N1	6093(6)	1540(8)	7041(2)	29.6(15)
N2	4529(6)	3168(8)	7301(2)	29.3(15)
N3	6637(6)	3101(7)	7803(2)	27.1(14)
N4	1204(6)	6483(7)	7947(2)	27.6(15)
N5	-425(6)	8129(8)	7696(2)	29.9(15)
N6	1589(6)	8086(8)	7197(3)	33.3(16)
C1	8044(7)	2611(9)	7893(3)	28.3(17)
C2	8385(9)	1716(10)	8340(3)	42(2)
C3	9815(10)	1350(11)	8467(4)	51(2)
C4	10817(10)	1883(12)	8142(4)	57(3)
C5	10519(8)	2740(11)	7698(4)	47(2)
C6	9121(8)	3138(11)	7570(3)	40(2)
C7	5816(8)	2618(9)	7389(3)	26.9(17)
C8	4969(7)	1452(10)	6621(3)	28.4(17)
C9	3781(8)	2317(11)	6880(3)	34.9(19)
C10	2972(8)	7640(9)	7093(3)	34.3(19)
C11	4138(9)	8175(11)	7418(4)	47(2)
C12	5488(9)	7810(11)	7280(5)	56(3)
C13	5697(11)	6972(13)	6813(5)	68(3)
C14	4613(11)	6432(12)	6506(4)	60(3)
C15	3233(10)	6757(10)	6642(4)	48(2)
C16	833(7)	7557(9)	7606(3)	24.6(17)
C17	-1106(8)	7237(11)	8113(3)	38(2)
C18	166(8)	6336(10)	8368(3)	34.1(19)
C19	-3251(14)	4078(15)	4682(4)	78(3)
C20	-2750(13)	5587(14)	4538(4)	71(3)
C21	-1948(12)	6450(13)	4994(4)	66(3)
C22	-2553(15)	7911(14)	5111(4)	81(4)
C23	-1508(15)	8851(15)	5485(5)	88(4)
C24	-2212(14)	10237(14)	5677(5)	91(4)
C25	-1278(10)	11252(11)	6026(4)	49(2)
C26	-661(9)	10418(11)	6517(3)	41(2)
C27	1560(11)	-692(13)	10399(4)	60(3)
C28	1781(10)	1000(12)	10426(3)	49(2)
C29	2016(9)	1752(10)	9881(3)	44(2)
C30	2919(9)	3135(11)	9920(3)	46(2)
C31	3066(9)	4045(11)	9398(3)	43(2)

C32	3998(8)	5452(11)	9471(3)	41(2)
C33	4078(8)	6392(10)	8964(3)	36(2)
C34	4594(8)	5483(9)	8492(3)	29.2(18)

Table S9. Anisotropic Displacement Parameters ($Å^2×10^3$) for ($Hclon^+$)(ala^-). The Anisotropic displacement factor exponent takes the form: $-2\pi^2[h^2a^{*2}U_{11}+2hka^*b^*U_{12}+…]$.

Atom	U_{11}	U_{22}	U_{33}	U_{23}	U_{13}	U_{12}
Cl1	42.6(14)	71.0(17)	50.2(14)	4.0(13)	8.2(10)	-3.4(12)
Cl2	74.4(17)	52.8(15)	45.4(13)	8.3(12)	-11.2(12)	-5.6(13)
Cl3	45.5(14)	63.4(16)	63.0(15)	6.9(13)	-13.9(11)	-2.9(12)
Cl4	82.6(18)	60.0(16)	51.3(14)	-3.7(13)	11.0(12)	0.9(14)
S1	39.2(12)	52.4(14)	35.2(11)	-4.0(11)	1.4(9)	10.8(11)
S2	49.8(15)	53.2(16)	56.1(15)	-10.5(12)	-0.6(12)	-2.3(11)
S3	81(2)	80(2)	53.9(15)	17.0(16)	-12.5(14)	7.9(17)
S4	81(2)	72(2)	55.3(16)	6.3(15)	4.2(14)	9.2(16)
O1	25(3)	46(4)	43(3)	2(3)	0(2)	19(3)
O2	36(3)	71(5)	63(4)	21(4)	11(3)	16(3)
O3	24(3)	43(3)	29(3)	-1(3)	2(2)	10(3)
O4	21(3)	60(4)	42(3)	-11(3)	-3(2)	15(3)
N1	16(3)	41(4)	32(3)	-5(3)	-5(3)	8(3)
N2	13(3)	44(4)	31(3)	-3(3)	-3(3)	14(3)
N3	18(3)	32(4)	31(3)	-7(3)	0(3)	12(3)
N4	15(3)	38(4)	30(3)	1(3)	2(3)	10(3)
N5	15(3)	43(4)	32(4)	4(3)	0(3)	17(3)
N6	19(3)	47(4)	35(4)	13(3)	-1(3)	14(3)
C1	15(3)	30(5)	39(4)	-9(3)	-8(3)	9(3)
C2	45(5)	40(5)	40(5)	-5(4)	-18(4)	13(4)
C3	56(5)	39(6)	56(6)	-11(4)	-30(4)	15(5)
C4	36(5)	59(7)	75(7)	-25(5)	-20(4)	17(5)
C5	21(4)	58(6)	62(6)	-27(4)	8(4)	10(4)
C6	23(4)	48(6)	48(5)	-14(4)	1(3)	8(4)
C7	22(4)	32(5)	26(4)	3(3)	-1(3)	8(3)
C8	19(4)	37(5)	29(4)	3(3)	-4(3)	6(3)
C9	22(4)	50(5)	32(4)	2(4)	-5(3)	10(4)
C10	20(4)	36(5)	47(4)	20(4)	11(3)	11(3)
C11	25(4)	41(5)	74(6)	25(4)	0(4)	12(4)
C12	19(4)	49(6)	101(7)	41(5)	4(5)	10(4)
C13	34(5)	63(7)	111(8)	47(6)	39(5)	22(5)
C14	63(6)	46(6)	73(7)	23(5)	40(5)	27(5)
C15	46(5)	48(6)	50(5)	16(4)	20(4)	15(4)
C16	12(3)	28(5)	34(4)	-2(3)	-4(3)	4(3)
C17	28(4)	50(5)	36(5)	-4(4)	7(3)	15(4)
C18	25(4)	52(6)	25(4)	2(4)	5(3)	2(4)
C19	90(9)	80(8)	63(6)	-19(6)	5(6)	5(6)
C20	86(8)	90(8)	36(5)	-3(5)	5(5)	10(6)
C21	62(7)	83(7)	53(6)	10(5)	4(5)	1(5)
C22	121(10)	77(7)	43(6)	11(5)	-9(6)	18(7)
C23	103(10)	95(9)	66(7)	-6(7)	-2(7)	28(7)
C24	114(10)	76(8)	80(8)	3(7)	-35(7)	38(7)
C25	46(4)	50(5)	52(4)	6(4)	5(3)	16(4)
C26	29(4)	46(6)	49(5)	2(4)	7(4)	22(4)

C27	57(6)	72(6)	51(5)	-7(5)	10(5)	-23(6)
C28	46(5)	60(5)	43(5)	1(4)	10(4)	2(5)
C29	41(5)	54(5)	36(5)	1(4)	-1(4)	10(4)
C30	46(5)	57(6)	36(5)	-8(4)	-1(4)	12(4)
C31	35(5)	53(6)	40(5)	-1(4)	-2(4)	5(4)
C32	24(4)	55(6)	42(4)	-3(4)	-1(4)	16(4)
C33	24(4)	47(5)	37(4)	-6(4)	-3(3)	17(4)
C34	25(4)	30(5)	33(4)	4(3)	2(3)	10(3)

Table S10. Bond Lengths for (Hclon$^+$)(ala$^-$).

Atom	Atom	Length/Å	Atom	Atom	Length/Å
Cl1	C6	1.715(9)	C1	C6	1.397(12)
Cl2	C2	1.722(10)	C2	C3	1.423(12)
Cl3	C11	1.706(10)	C3	C4	1.350(14)
Cl4	C15	1.715(11)	C4	C5	1.349(14)
S1	C29	1.847(9)	C5	C6	1.402(11)
S1	S2	2.056(3)	C8	C9	1.524(11)
S2	C27	1.811(9)	C10	C15	1.382(12)
S3	C21	1.853(11)	C10	C11	1.425(12)
S3	S4	2.052(5)	C11	C12	1.380(12)
S4	C19	1.789(11)	C12	C13	1.382(16)
O1	C26	1.249(10)	C13	C14	1.343(16)
O2	C26	1.250(10)	C14	C15	1.398(13)
O3	C34	1.263(9)	C17	C18	1.561(11)
O4	C34	1.255(9)	C19	C20	1.464(17)
N1	C7	1.311(10)	C20	C21	1.535(15)
N1	C8	1.461(9)	C21	C22	1.446(15)
N2	C7	1.329(9)	C22	C23	1.567(18)
N2	C9	1.443(10)	C23	C24	1.481(11)
N3	C7	1.329(9)	C24	C25	1.508(16)
N3	C1	1.418(9)	C25	C26	1.513(12)
N4	C16	1.306(9)	C27	C28	1.512(14)
N4	C18	1.460(9)	C28	C29	1.516(12)
N5	C16	1.327(9)	C29	C30	1.496(13)
N5	C17	1.461(10)	C30	C31	1.521(12)
N6	C16	1.338(10)	C31	C32	1.535(13)
N6	C10	1.407(10)	C32	C33	1.498(11)
C1	C2	1.381(11)	C33	C34	1.506(11)

Table S11. Bond Angles for (Hclon+)(ala-).							
**Atom**	**Atom**	**Atom**	**Angle/°**	**Atom**	**Atom**	**Atom**	**Angle/°**
C29	S1	S2	90.6(3)	C14	C13	C12	121.5(9)
C27	S2	S1	92.3(3)	C13	C14	C15	120.3(10)
C21	S3	S4	94.2(4)	C10	C15	C14	120.3(10)
C19	S4	S3	90.6(5)	C10	C15	Cl4	119.7(7)
C7	N1	C8	109.7(6)	C14	C15	Cl4	120.0(8)
C7	N2	C9	110.7(7)	N4	C16	N5	113.3(7)
C7	N3	C1	123.1(6)	N4	C16	N6	126.3(6)
C16	N4	C18	110.0(6)	N5	C16	N6	120.4(7)
C16	N5	C17	109.7(6)	N5	C17	C18	101.4(6)
C16	N6	C10	124.8(6)	N4	C18	C17	102.0(6)
C2	C1	C6	118.9(7)	C20	C19	S4	108.3(8)
C2	C1	N3	119.5(7)	C19	C20	C21	115.8(9)
C6	C1	N3	121.3(7)	C22	C21	C20	113.4(10)
C1	C2	C3	120.0(9)	C22	C21	S3	112.0(8)
C1	C2	Cl2	120.3(6)	C20	C21	S3	106.5(8)
C3	C2	Cl2	119.6(7)	C21	C22	C23	109.9(10)
C4	C3	C2	118.9(9)	C24	C23	C22	109.8(11)
C5	C4	C3	122.6(9)	C23	C24	C25	114.0(10)
C4	C5	C6	119.4(9)	C24	C25	C26	111.4(9)
C1	C6	C5	120.1(8)	O1	C26	O2	124.4(8)
C1	C6	Cl1	120.3(6)	O1	C26	C25	118.1(7)
C5	C6	Cl1	119.6(7)	O2	C26	C25	117.4(8)
N1	C7	N2	111.5(7)	C28	C27	S2	109.2(7)
N1	C7	N3	127.0(7)	C27	C28	C29	114.8(8)
N2	C7	N3	121.3(7)	C30	C29	C28	113.9(8)
N1	C8	C9	102.4(6)	C30	C29	S1	111.6(6)
N2	C9	C8	101.8(6)	C28	C29	S1	106.3(6)
C15	C10	N6	120.0(8)	C29	C30	C31	116.6(7)
C15	C10	C11	118.4(7)	C30	C31	C32	113.6(7)
N6	C10	C11	121.4(8)	C33	C32	C31	113.5(7)
C12	C11	C10	120.0(10)	C32	C33	C34	111.6(7)
C12	C11	Cl3	120.5(8)	O4	C34	O3	124.1(7)
C10	C11	Cl3	119.5(6)	O4	C34	C33	117.0(7)
C11	C12	C13	119.5(10)	O3	C34	C33	118.8(6)

Table S12. Hydrogen Bonds for $(Hclon^+)(ala^-)$.

D	H	A	d(D-H)/Å	d(H-A)/Å	d(D-A)/Å	D-H-A/°
N1	H1	O1[1]	0.86	1.90	2.726(8)	161.8
N2	H2	O3	0.86	1.95	2.756(8)	155.5
N3	H3	O4	0.86	1.75	2.600(8)	171.8
N4	H4A	O3	0.86	1.89	2.711(7)	160.0
N5	H5A	O1	0.86	1.97	2.763(8)	152.6
N6	H6	O2	0.86	1.75	2.602(9)	172.8
C14	H14	S4[2]	0.93	2.93	3.821(10)	161.1
C20	H20B	O2[3]	0.97	2.41	3.380(12)	178.2
C25	H25A	Cl1[4]	0.97	2.93	3.640(10)	131.2
C28	H28A	O4[5]	0.97	2.64	3.466(11)	143.5

Table S13. Hydrogen Atom Coordinates (Å×10^4) and Isotropic Displacement Parameters (Å^2×10^3) for $(Hclon^+)(ala^-)$.

Atom	*x*	*y*	*z*	U(eq)
H1	6826.2	971.84	7058.33	35
H2	4188.51	3927.43	7471.28	35
H3	6298.39	3741.15	8027.88	32
H4A	1949.96	5940.8	7926.94	33
H5A	-785.61	8906.75	7533.67	36
H6	1202.11	8746.98	6982.89	40
H3A	10053.84	752.56	8768.95	61
H4	11750.65	1650.38	8228.88	69
H5	11234.69	3064.19	7477.13	56
H8A	4704.64	410.3	6546.26	34
H8B	5243.26	1928.55	6284.3	34
H9A	3303.54	2979.55	6618.46	42
H9B	3103.58	1634.54	7034.57	42
H12	6250.63	8124.02	7498.51	68
H13	6608.57	6778.49	6708.96	82
H14	4780.88	5838.18	6201.88	72
H17A	-1812.33	6564.74	7953.19	46
H17B	-1533.2	7879.25	8382.12	46
H18A	500.92	6781.72	8710.29	41
H18B	-76.22	5284.8	8428.67	41
H19A	-2515.91	3336.14	4632.77	94
H19B	-4053.79	3803.27	4447.34	94
H20A	-3552.35	6190.82	4417.05	85
H20B	-2141.7	5488.43	4230.4	85
H21	-981.74	6606.84	4881.94	79
H22A	-3431.6	7775.13	5292.51	97
H22B	-2747.16	8452.13	4771.94	97
H23A	-1197.08	8244.34	5796.84	106
H23B	-690.28	9124.52	5282.52	106
H24A	-3015.78	9944.94	5885.23	109
H24B	-2560.65	10804.57	5361.32	109
H25A	-1817.9	12110.83	6147.55	59
H25B	-522.8	11633.86	5808.74	59
H27A	733.37	-965.99	10596.73	72
H27B	2363.67	-1207.73	10566.78	72
H28A	2586.58	1205.82	10668.54	59

H28B	966.97	1458.35	10586.09	59
H29	1100.04	2042.65	9718.78	52
H30A	3851.08	2830.73	10049.79	55
H30B	2542.7	3797.61	10194.64	55
H31A	2139.59	4358.32	9265.31	51
H31B	3458.3	3397.56	9122.42	51
H32A	3636.12	6071.01	9761.12	49
H32B	4938.93	5133.6	9584.04	49
H33A	4706.98	7237.94	9034.52	43
H33B	3154.1	6795.75	8869.42	43
